# Multi-target measurable residual disease assessed by error-corrected sequencing in patients with acute myeloid leukemia: An ALFA study

**DOI:** 10.1038/s41408-024-01078-8

**Published:** 2024-06-13

**Authors:** Pierre Hirsch, Jérôme Lambert, Maxime Bucci, Caroline Deswarte, Augustin Boudry, Juliette Lambert, Laurene Fenwarth, Jean-Baptiste Micol, Christine Terré, Karine Celli-Lebras, Xavier Thomas, Hervé Dombret, Nicolas Duployez, Claude Preudhomme, Raphael Itzykson, Francois Delhommeau

**Affiliations:** 1 Sorbonne Université, INSERM, Centre de Recherche Saint-Antoine, CRSA, AP-HP, SIRIC CURAMUS, Hôpital Saint-Antoine, Service d’Hématologie Biologique, 75012 Paris, France; 2 Biostatistics and Medical Information Department, Hôpital Saint Louis, Paris, France; 3 INSERM U1153 - ECSTRRA Team, Hôpital Saint Louis, Paris, France; 4 Univ. Lille, CNRS, Inserm, CHU Lille, UMR9020-U1277 - CANTHER - Cancer Heterogeneity Plasticity and Resistance to Therapies, F-59000 Lille, France; 5 Service d’Hématologie Clinique, André Mignot Hospital, Le Chesnay, France; 6 Gustave Roussy, Université Paris-Saclay, Villejuif, France; 7 Laboratory of Hematology, André Mignot Hospital, Le Chesnay, France; 8 ALFA Group, Paris, France; 9 Lyon Sud, University Hospital, 69495 Pierre-Bénite, Lyon, France; 10 Département Hématologie et Immunologie, Hôpital Saint-Louis, Assistance Publique-Hôpitaux de Paris, F-75010 Paris, France; 11 Université Paris Cité, Génomes, biologie cellulaire et thérapeutique U944, INSERM, CNRS, F-75010 Paris, France

**Keywords:** Cancer genetics, Acute myeloid leukaemia

## Abstract

The evaluation of measurable residual disease (MRD) in acute myeloid leukemia (AML) using comprehensive mutation analysis by next-generation sequencing (NGS) has been investigated in several studies. However controversial results exist regarding the detection of persisting mutations in *DNMT3A*, *TET2*, and *ASXL1* (DTA). Benchmarking of NGS-MRD taking into account other molecular MRD strategies has to be done. Here, we performed error-corrected-NGS-MRD in 189 patients homogeneously treated in the ALFA-0702 study (NCT00932412). Persistence of non-DTA mutations (HR = 2.23 for RFS and 2.26 for OS), and DTA mutations (HR = 2.16 for OS) were associated with poorer prognosis in multivariate analysis. Persistence of at least two mutations in complete remission (CR) was associated with a higher cumulative incidence of relapse (CIR) (HR = 3.71, *p* < 0.0001), lower RFS (HR = 3.36, *p* < 0.0001) and OS (HR = 3.81, *p* = 0.00023) whereas persistence of only one mutation was not. In 100 analyzable patients, *WT1*-MRD, but not NGS-MRD, was an independent factor for RFS and OS. In the subset of 67 *NPM1* mutated patients, both *NPM1* mutation detection (*p* = 0.0059) and NGS-MRD (*p* = 0.035) status were associated with CIR. We conclude that detectable NGS-MRD including DTA mutations correlates with unfavorable prognosis in AML. Its integration with alternative MRD strategies in AML management warrants further investigations.

## Introduction

Acute myeloid leukemia (AML) is an aggressive malignancy that emerges from the accumulation of genetic events in hematopoietic stem or early progenitor cells [1–3]. The mutational landscape of AML has been largely unraveled [4–6]. Recurrent chromosomal and molecular abnormalities define distinct AML subtypes [1, 5, 7, 8], and allow prognostic stratification [9, 10].

Measurable residual disease (MRD), *i.e*. detection of persistent AML cells below the cytological level of detection, is also a major prognostic factor for relapse after chemotherapy. Many laboratory tools have been developed for MRD detection. Direct detection of persisting AML cells by flow cytometry has been thoroughly validated but is limited by its sensitivity and applicability [11–13]. Molecular MRD consists of the detection or quantification after treatment of AML-associated genetic alterations. For recurrent gene fusions [14, 15] or *NPM1* mutations [16–18], molecular MRD evaluation by quantitative PCR is also standard practice [12]. These markers can be used with high sensitivity (detection threshold 10^−4^ to 10^−5^) and specificity but in only half of AML cases [6]. Conversely, *WT1* expression is a more universal MRD marker (overexpressed in 70–90% AML). Despite its lower threshold of detection (at most 10^−3^), the persistence of a high *WT1* expression in complete remission (CR) is associated with shorter RFS and OS [19–21].

NGS-based strategies can also be used for MRD assessment. Initial studies focused on only one or a few genes such as *IDH1/2*, *DNMT3A*, *RUNX1*, or others [22, 23] and showed the proof-of-concept for NGS-based MRD assessment. Multi-target detection using NGS (NGS-MRD) has also been proposed. The first studies used a relatively high detection threshold (1 to 5% of mutant allele) with standard NGS (stNGS) [24, 25]. More recent studies used error-corrected NGS, allowing a lower threshold of detection (10^−3^ to 10^−5^), with either specific panels including all mutations detected at diagnosis, or panels including only a little selection of genes [26–30]. These studies suggested an interest of NGS-MRD. Overall, the detection of one or more mutations after intensive chemotherapy was independently associated with higher cumulative incidence of relapse (CIR), and with lower relapse-free survival (RFS) and overall survival (OS) probabilities, with conflicting data for mutations in *DNMT3A*, *TET2* or *ASXL1* (DTA) whose persistence is considered by some authors to be a “return” to pre-leukemic hematopoiesis state without prognostic value [26, 28, 31].

Several studies compared NGS-MRD to flow cytometry MRD (MFC) [26, 28, 32, 33], but the benefit of NGS-MRD when compared to other molecular MRD markers has not been investigated.

The goal of the present study was to evaluate NGS-MRD strategy in the ALFA-0702 study, with a clonal architecture-based interpretation, focusing on the value of DTA and multiple mutation detection in CR. We also aimed to investigate the added value of NGS-MRD as compared to *NPM*1- and *WT1*-MRD.

## Material, subjects, and methods

### Patients, material, and treatments

The ALFA-0702 study (NCT00932412) enrolled 713 patients aged 18–60 years with newly diagnosed de novo AML and excluded acute promyelocytic leukemia, core binding factor AML, and Philadelphia chromosome–positive AML. All patients received the same induction chemotherapy [34]. Patients in CR received either allo-SCT or were randomly assigned for high-dose cytarabine (HDAC) or clofarabine plus cytarabine (CLARA) consolidation chemotherapy. The protocol was approved in December 2008 by the Institutional Review Board of the French Regulatory Agency and the Ethics Committee Sud-Est IV. All patients gave informed consent for both treatment and genetic analysis, according to the Declaration of Helsinki. The median follow-up time was 49 months after CR.

All peripheral blood (PB) and bone marrow (BM) samples were prospectively collected at the time of inclusion and at the evaluation of the response to induction chemotherapy (days 28 to 45).

#### *Cytogenetic analysis and FLT3*-ITD screening

Cytogenetic R-banding analysis was performed on diagnostic BM samples using standard methods. Karyotypes were reported according to the International System for Human Cytogenetic Nomenclature recommendations [9]. *FLT3*-internal tandem duplication (*FLT3*-ITD) was assessed centrally on genomic DNA, as previously described [35]. All patients were classified according to ELN2017 recommendations [10].

### NGS at AML diagnosis

DNA was extracted from diagnosis BM samples. A 67 gene panel was designed (supplementary table 1). Libraries were obtained from 200 ng of DNA, using custom myeloid solution (Sophia Genetics) and according to the manufacturer protocol. Sequencing was performed using a Nextseq sequencer (Illumina). Alignments and variant calling were performed with a dedicated bioinformatics pipeline using the Sophia DDM software (Sophia Genetics). Sequencing depth was over 500x in ≥ 95% targeted regions in 95% of samples.

### Error corrected NGS in remission

DNA was extracted from post-induction BM samples. A specific panel was designed for MRD detection including all mutations identified at diagnosis. Libraries were prepared by a capture method (Twist Bioscience®) with xGen UDI-UMI adapter (IDT®) using 500 ng DNA. Samples were sequenced with NovaSeq Illumina®. The base calling was performed with bcl2fastq2 (v:2.20.0) and fastq was trimmed with fastp (v:0.20.0) and aligned with bwa mem (v:0.7.17). The calling of consensus reads was performed with fgbio (v:1.0.0) setting the minimum number of reads to produce a consensus base to one. The median depth on variants after deduplication was 24 695x (range 1 378 to 69 913x). All variants were manually checked with IGV software to evaluate their persistence in CR samples. Variants of unknown significance (VUS) at diagnosis and with variant allele frequency (VAF) in CR around 50% were considered as germline variants and were excluded from further analyses. Even if the threshold of detection was lower in most targets, a consensual threshold of 0.1% was set for NGS-MRD detection. This threshold was proposed for a simple interpretation without dependence on local depth. Some FLT3-ITD were detected at diagnosis only with standard PCR and not with NGS (*n* = 10) and could have been misevaluated in CR due to alignment failure. For a few other targets (supplementary table 4), only semi-quantitative quantification could be done due to high local background noise.

For specific *NPM1* mutation MRD analysis, the median local depth was 18 468x. Consequently in the specific *NPM1* and NGS MRD benchmarking section, the detection of any mutated consensus read in *NPM1* in CR was considered as positivity, allowing a theoretical threshold for MRD detection of at least 10^−4^. NGS *NPM1* and published RT-PCR *NPM1*-MRD data [18] were not compared.

### Quantification of WT1 expression levels

The quantification of *WT1* transcripts was performed as already described [36]. Briefly, *WT1* mRNA levels were normalized to the *ABL1* control gene. Results were expressed as the ratio *WT1* copy number/*ABL1* copy number × 100. The upper limit of normal was defined as 2.5% in BM samples or as 0.5% in PB samples. Testing for *WT1* was performed in the same BM sample as the one used for NGS-MRD, or alternatively in a PB sample collected the same day.

### Statistical analyses

Qualitative variables are presented as counts and percentages and quantitative variables as median and range. Comparisons of patient characteristics between groups were performed using Fisher’s exact test, Kruskall Wallis, or Mann-Whitney test as appropriate. Complete remission (CR) was defined as recovery of morphologically normal BM and normal blood count, and CR with incomplete blood recovery (CRi) as recovery of morphologically normal BM with persistent cytopenia. Overall survival (OS) was defined as the time between the date of diagnosis and death. Relapse-free survival (RFS) was defined as the time between the date of CR1 and the date of the first relapse or death. Data were not censored at the time of allo-SCT. OS and RFS were estimated using the Kaplan-Meier estimate and differences were tested with log-rank test. Cumulative incidence of relapse (CIR) was estimated within a competing-risk framework, with death without relapse as a competing endpoint. The independent prognostic value of MRD was assessed by fitting multivariable Cox models for all 3 outcomes. Results are presented as hazard ratios (HR) [95% confidence intervals].

The impact of allo-SCT on the risk of relapse or death without relapse, cause-specific risk of relapse, and risk of death was assessed in the subgroup of patients with intermediate-/unfavorable-risk AML only, using allo-SCT as a time-dependent covariate. The interaction between the prognostic effect of MRD and the effect of allo-SCT was assessed by adding an interaction term in the Cox multivariable model. For all analyses, *p*-values under 0.05 were considered statistically significant.

## Results

### Patient characteristics

**Fig. 1:**
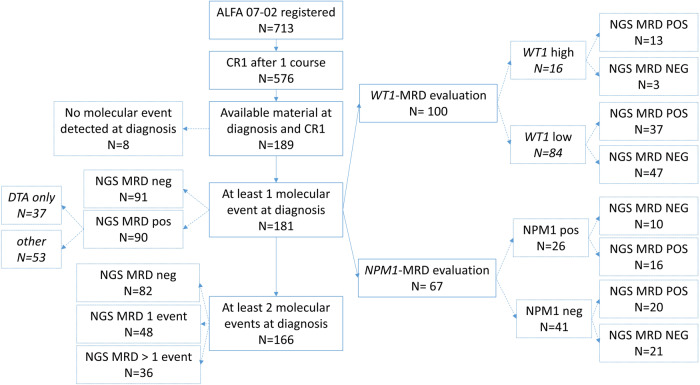
Flow chart of the study. CR1: Complete remission or complete remission with incomplete recovery; DTA: mutations in *DNMT3A*, *TET2*, or *ASXL1*; NGS-MRD: measurable residual disease using Next Generation Sequencing assay with a 0.1% threshold for positivity; neg: negative; pos: positive; *NPM1*-MRD: measurable residual disease for *NPM1* mutation measured by Next Generation sequencing assays with a threshold of detection over 10^−4^ (see methods).

713 patients were included in the ALFA-0702 study and 576 reached CR/CRi in one course. Only 189 patients who reached CR (*n* = 179) or CRi (*n* = 10) after the first induction course (henceforth CR1) had available material for NGS analysis at both time points (Fig. 1). Comparison between the 189 patients and the 387 remaining patients with no material in CR was performed (supplementary table 2). Patients of the present study were more likely to harbor adverse or intermediate ELN risk (*p* = 0.043). There were no other major differences with other patients, and 2-years outcome was the same between the groups (PFS, OS, and CIR). (supplementary table 3). Eight patients (4%) had no identified molecular marker detected at diagnosis and could not be analyzed. Out of the 181 remaining patients, 15 had only one identified mutation, and 166 had at least two mutations. The main patient characteristics are described in Table 1, Supplementary Tables 4 and 5. Median age was 46 years old (18–60), and median leukocyte count was 7.9 × 10^9^/L (0.5–256). Karyotype was normal in 104 patients. ELN 2017 risk distribution was favorable in 52 (29%) patients, intermediate in 71 (40%), and adverse in 56 (31%). All patients received cytarabine consolidation courses, with (*n* = 73, 40%) or without (*n* = 108, 60%) clofarabine. Ninety-two (92) of the 127 intermediate/adverse risk patients received allo-SCT in CR1.

**Table 1:** Major patients’ characteristics.

		Total	NGS^other^	NGS^DTA^	NGS^NEG^	P val
		(*n* = 181)	(*n* = 53)	(*n* = 37)	(*n* = 91)	
**AGE, yo : median (range)**		46 (18–60)	48 (24–60)	48 (30–60)	42 (18–60)	0.0063
**GENDER : Male n (%)**		99 (55)	27 (51)	17 (46)	55 (60)	NS
**ELN 2017**^a^ **: N (%)**	FAV	52 (29)	10 (20)	14 (38)	28 (31)	0.08
	INT	71 (40)	18 (35)	16 (43)	37 (41)	
	ADV	56 (31)	23 (45)	7 (19)	26 (28)	
**Leukocytes, x10**^**9**^**/L**		7.9 (0.5–256)	8.9 (1–126)	17 (1.4–256)	6.77 (0.5–249)	0.054
**N mutations (diag) :** **n(range)**		4 (1–10)	4 (1–10)	5 (2–10)	3 (1–9)	0.0009
**N mutations (CR1) :** **n(range)**		1 (0–7)	2 (1–6)	1 (1–7)	0	<0.0001
**CLARA (consolidation)**		73 (40)	25 (47)	17 (49)	31 (34)	NS
**allo-SCT in CR1**^b^		92 (72)	31 (75)	16 (69)	45 (71)	NS

^a^2 missing.

^b^ELN2017 fav excluded.

*NS* not significant, *FAV* favorable, *INT* intermediate, *ADV* adverse, diag diagnosis, *NGS*^*NEG*^ no mutation detected in CR1, *NGS*^*DTA*^ detection of at least one mutation in CR1 only in *DNMT3A*, *TET2* or *ASXL1,* *NGS*^*other*^ detection of at least one mutation in CR1 in any other gene than *DNMT3A*, *TET2* or *ASXL1*.

### Mutational profile at diagnosis and CR1

**Fig. 2:**
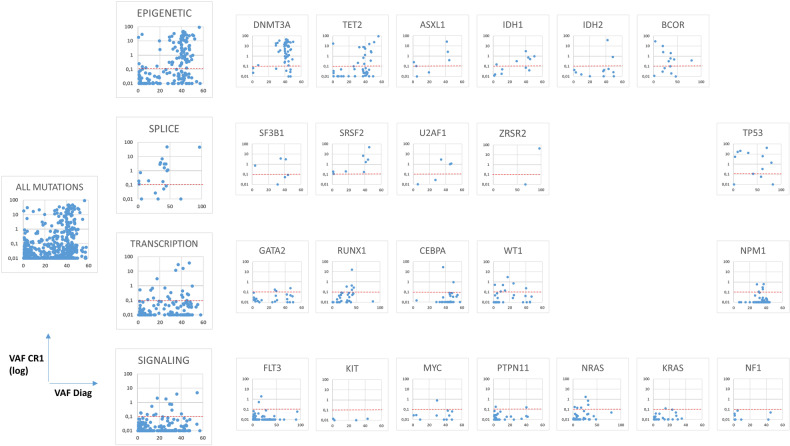
Mutational profile at diagnosis and CR1 according to functional categories and gene mutations. Diagnosis variant allele frequency (VAF) is plotted in x-axis (range 0–60% or 0–100% for genes with loss of heterozygosity) and VAF in CR1 in the y-axis (log-scale). The dotted red-line represents the retained threshold of detection of 0.1%. Mutations with VAF in CR1 under 0.01% were plotted at 0.01%. The “Epigenetic” plot summarizes data on *DNMT3A*, *TET2*, *ASXL1 IDH1*, *IDH2*, *BCOR and ASXL2*, *ATRX*, *BCORL1*, *CTCF*, *CUX1*, *EP300*, *EZH2*, *KDM6A*, *KMT2C*. The “Splice” plot summarizes data on *SF3B1*, *SRSF2*, *U2AF1*, and *ZRSR2*. The “Transcription” plot summarizes data on *GATA2*, *RUNX1*, *CEBPA*, *WT1*, *PHF6*, *ETV6*, and *IKZF1*. The “signaling” plot summarizes data on *FLT3*, *KIT*, *MYC*, *PTPN11*, *NRAS*, *KRAS*, *NF1* and *JAK2*, *JAK3*, *CHEK2*, *CSF3R*, *CBL* and *BRAF*. The “All mutations” plot contains combined data of all mutated genes. A few mutations with uncertain VAFs at diagnosis or CR1 were not plotted (see methods).

A total of 735 somatic mutations were identified at diagnosis in 181 patients. The median number of gene mutations was 4 (range 1–10). Four potential germline mutations (in *CEBPA*, *NF1*, *DDX41*, and *RUNX1*) were excluded from all analyses. Twenty-three VUS with persistence at a high level in CR were identified. As patients with only VUS persistence in CR1 had the same prognosis as negative MRD patients (not shown), VUS were excluded from further analyses. Detailed mutational data at diagnosis and CR1 are described in Fig. 2, supplementary Fig. 1, and Supplementary Table 4. The most frequently mutated genes detected were *FLT3* (*n* = 56 ITDs and *n* = 52 other mutations), *NPM1* (*n* = 67), *NRAS* (*n* = 57), *DNMT3A* (*n* = 50), and *TET2* (*n* = 45).

The persistence of mutations in CR1 was variable according to gene’s identity and function. Regarding epigenetic regulators, some mutations were often detected in CR1, in particular mutations in *DNMT3A* (*n* = 42/50), *IDH1* (*n* = 6/12), or *BCOR* (*n* = 9/15). This was less frequent for mutations in *TET2* (*n* = 15/45) and was uncommon for mutations in some other genes such as *IDH2* (*n* = 2/13) or *EZH2* (*n* = 1/8). Other frequently persisting events included mutations in *TP53* (*n* = 9/12) or in splice machinery components such as *SRSF2* (*n* = 8/8) and *U2AF1* (*n* = 3/5). Mutations in hematopoietic transcription factors, *NPM1*, or signal transduction-associated genes were infrequently detected in CR1 with the 0.1% threshold (Fig. 2 and supplementary Fig. 1).

Ninety-one patients had no mutation detected in CR1 (NGS^NEG^), and 90 had at least one mutation detectable, including 37 with only *DTA* mutations (NGS^DTA^) and 53 with at least one other gene mutation (NGS^other^). When comparing the main characteristics of the 3 groups (Table 1), NGS^NEG^ patients were significantly younger (*p* = 0.0063). There was also a trend for the ELN2017 distribution to be different, with more favorable risk patients in the NGS^DTA^ group, more adverse risk patients in the NGS^other^ group (*p* = 0.08), and a trend for the NGS^DTA^ group to harbor a higher initial leukocyte count (*p* = 0.054).

When separating patients according to Lindsley classification [37], NGS-MRD group was highly associated with AML ontogeny (*p* < 0.0001). De novo disease ontogeny was over-represented in the NGS^DTA^ group (mainly due to *NPM1* association), and under-represented in the NGS^other^ group. In contrast, *TP53* and secondary ontogeny genes were over-represented in the NGS^other^ group (Supplementary Table 6).

### NGS-MRD including DTA mutations is associated with poor prognosis

**Fig. 3:**
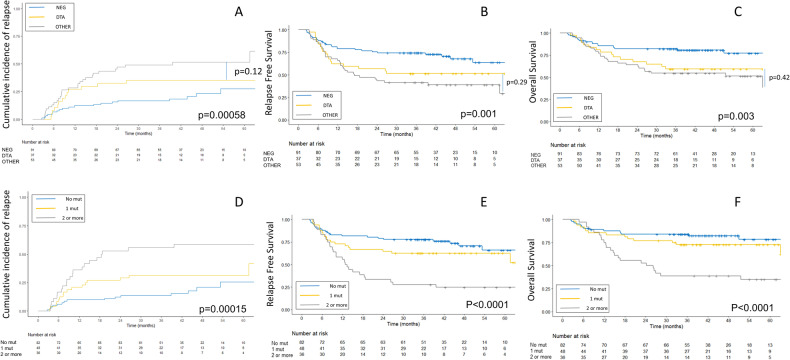
Prognosis according to NGS-MRD. Prognosis according to NGS status (**A**–**C**) and the number of persistent mutations (**D**–**F**). *p*-values are for log-rank tests for Relapse Free Survival and Overall Survival and for Gray test for Cumulative incidence of Relapse. Data were not censored at allogeneic hematopoietic stem cell transplantation. NEG no mutation detected in CR1, DTA detection of only *DNMT3A*, *TET2* or *ASXL1* mutation in CR1; other: detection of other mutation than *DNMT3A*, *TET2* or *ASXL1* in CR1.

We first analyzed the prognosis of the 3 groups of patients (NGS^NEG^, NGS^DTA^, and NGS^other^). CIR was found significantly different between the three groups, with probabilities of 23% [13–33], 35% [19–51], and 51% [38–66] at 24 months for NGS^NEG^, NGS^DTA^, and NGS^other^ groups, respectively (*p* = 0.0003). At 4 years, RFS estimates were 68% [58–80], 51%[38–70] and 39% [28–55] (*p* = 0.001), and OS estimates were 80% [73–89], 59%[46–78] and 54% [43–70] (*p* = 0.003), respectively, with prolonged survival in the NGS^NEG^ group (Fig. 3A–C; Supplementary table 7). No significant differences were found when comparing CIR, RFS, and OS between NGS^other^ and NGS^DTA^ groups. The proportion of patients receiving CLARA as post-CR1 therapy was the same in the 3 groups.

In multivariate models adjusted with ELN2017 status and age, only NGS^other^ was associated with higher CIR (HR = 2.73[1.48–5.03], *p* = 0.0013) when the trend for higher CIR in NGS^DTA^ did not reach statistical significance (HR = 1.77[0.86–3.64], *p* = 0.12). NGS^other^ (HR = 2.23[1.31–3.81], *p* = 0.0033) was predictive of worse RFS, but NGS^DTA^ (HR = 1.79[0.97–3.30], *p* = 0.062) and ELN2017 did not reach statistical significance (*p* = 0.074). Both adverse ELN2017 risk (HR = 2.64[1.25–5.56], *p* = 0.011), NGS^DTA^ (HR = 2.16[1.07–4.37], *p* = 0.032), and NGS^other^ (HR = 2.26[1.21–4.24], *p* = 0.011) were associated with OS (Table 2).

**Table 2:** Multivariable analysis for cumulative incidence of relapse, relapse free survival and overall survival according to NGS MRD status.

	CIR			RFS			OS		
	HR	CI	*P*-value	HR	CI	*P*-value	HR	CI	*P*-value
**NGS-MRD (neg)**	1			1			1		
**NGS MRD (DTA)**	1.77	0.86–3.64	0.12	1.79	0.97–3.30	0.062	2.16	1.07–4.37	0.032
**NGS MRD (other)**	2.73	1.48–5.03	0.0013	2.23	1.31–3.81	0.0033	2.26	1.21–4.24	0.011
**ELN2017 (FAV)**	1			1			1		
**ELN2017 (INT)**	1.78	0.90–3.53	0.10	1.63	0.89–2.97	0.11	1.70	0.80–3.59	0.17
**ELN2017 (ADV)**	1.62	0.78–3.35	0.19	1.77	0.95–3.31	0.074	2.64	1.25–5.56	0.011
**Age**	1.01	0.99–1.04	0.33	1.01	0.99–1.04	0.46	1.01	0.99–1.04	0.33

*NS* not significant, *HR* Hazard ratio, *CI* 95% confidence interval, *FAV* favorable, *INT* intermediate, *ADV* adverse, *CIR* cumulative incidence of relapse, *RFS* relapse-free survival, *OS* overall survival.

### The number of NGS-MRD persisting events is associated with prognosis

As the detection of only one molecular event at MRD could be linked with CHIP-related pre-leukemic clones with blunted relapse-initiating capacity, we next investigated whether the persistence of multiple mutations (including DTA mutations) was associated with prognosis. All 166 patients with at least two gene mutations at diagnosis (Fig. 1) were included. Eighty-two patients had no mutation detected in CR (NGS^null^), 48 had only one mutation (NGS^one^), and 36 had two mutations or more (NGS^more^). Patients in the NGS^DTA^ group harbored more often 1 mutation in CR than patients in NGS^other^ (76% vs. 46%, respectively, *p* = 0.0099) (Table 1 and Supplementary Table 8).

CIR was significantly different when comparing patients from NGS^null^, NGS^one^, or NGS^more^ groups, with 48-month estimates at 20%[10–31], 31%[18–45], and 58%[42–75], respectively (*p* = 0.0012). Likewise, at 48 months, probabilities of RFS were 70% [60–83], 62% [50–78], and 24%[14–44], respectively (*p* < 0.0001), and probabilities of OS were 82%[74–91], 72%[61–87], and 38%[26–59], respectively (*p* < 0.0001) (Fig. 3D–F). In univariate analysis of both RFS and OS, NGS^one^ group was not significantly different from NGS^null^ group (HR = 1.66[0.91–3.04] and HR = 1.72[0.84–3.51] respectively).

In multivariate models adjusted with ELN2017 status and age, NGS^one^ was marginally associated with higher CIR (HR = 1.92[0.94–3.92], *p* = 0.072), but was not associated with RFS nor OS. Conversely, NGS^more^ was associated with higher CIR (HR = 3.71[1.82–7.56], *p* < 0.0001) and shorter RFS (HR = 3.36[1.83–6.17], *p* < 0.0001) and OS (HR = 3.81[1.87–7.74], *p* = 0.00023) (Table 3).

**Table 3:** Multivariable analysis for cumulative incidence of relapse, relapse free survival and overall 608 survival including to the number of persisting mutations.

	CIR			RFS			OS		
	HR	CI	*P*-value	HR	CI	*P*-value	HR	CI	*P*-value
**NGS MRD neg**	1			1			1		
**NGS MRD 1 mut**	1.92	0.94–3.92	0.072	1.58	0.85–2.95	0.15	1.60	0.77–3.35	0.21
**NGS MRD 2 or more mut**	3.71	1.82–7.56	< 0.0001	3.36	1.83–6.17	< 0.0001	3.81	1.87–7.74	0.00023
**ELN2017 FAV**	1			1			1		
**ELN2017 INT**	1.78	0.89–3.57	0.10	1.60	0.87–2.96	0.13	1.76	0.83–3.75	0.14
**ELN2017 ADV**	1.36	0.63–2.94	0.43	1.53	0.80–2.93	0.20	2.26	1.05–4.87	0.037
**Age**	1.01	0.98–1.04	0.57	1.00	0.98–1.03	0.88	1.00	0.87–1.03	0.89

*HR* Hazard ratio, *CI* 95% confidence interval, *FAV* favorable, *INT* intermediate, *ADV* adverse, *CIR* cumulative incidence of relapse, *RFS* relapse-free survival, *OS* overall survival.

### Comparison of NGS-MRD and WT1-MRD

**Fig. 4:**
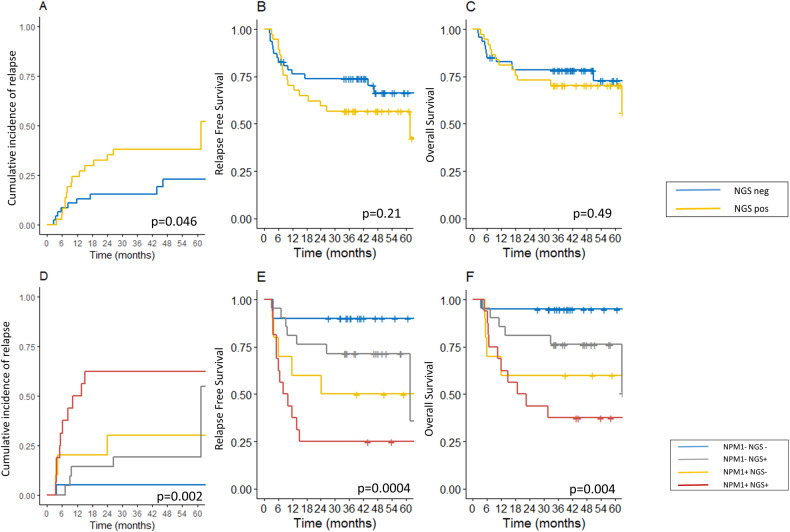
Prognosis of NGS-MRD evaluation when compared to *WT1* expression in CR and NPM1 MRD. Panels **A**–**C** represent the cumulative incidence of relapse, relapse-free survival, and overall survival according to NGS-MRD in CR1 status in the 84 patients with low *WT1* expression in CR1. Panels **D–F** represent the cumulative incidence of relapse, relapse-free survival, and overall survival according to NGS-MRD in CR1 and *NPM1* MRD assessed by NGS in the 67 *NPM1* mutated patients. *p*-values are for the log-rank test for RFS and OS and for the Gray test for CIR. Data were not censored at allogeneic hematopoietic stem cell transplantation.

We then investigated whether NGS-MRD could add prognostic information when compared to other validated MRD strategies. In 100 patients with *WT1* overexpression at baseline, both *WT1* expression and NGS-MRD data were available in CR1 (Fig. 1). Eighty-four patients had low *WT1* expression in CR1 (WT1^low^) including 37 with at least one marker detected by NGS-MRD (NGS^POS^-19 patients with only DTA and 8 with multiple mutations) and 47 with no marker detected by NGS-MRD (NGS^NEG^). Sixteen patients harbored high *WT1* expression at CR1 (WT1^high^) including 13 with NGS^POS^, and 3 with NGS^NEG^ (Fig. 1, supplementary Fig. 2). WT1^high^ was associated with higher CIR at 4 years (*p* = 0.005) and shorter RFS (*p* = 0.0008) with a trend toward shorter OS (*p* = 0.06) (supplementary Fig. 3). We consequently focused on the WT1^low^ patients. In these patients, NGS^POS^ was associated with higher CIR at 4 years (37% [22–54] vs. 22% [9–37] *p* = 0.04) but was not associated with RFS nor OS. (Fig. 4A–C). In multivariable analysis including NGS-MRD, *WT1*-MRD, and ELN2017, only *WT1*-MRD status was associated with RFS (HR = 3.21 [1.55–6.67], *p* = 0.0017) and OS (HR = 2.71 [1.13–6.49], *p* = 0.0025) (Supplementary Table 9).

### Comparison of multi-target NGS-MRD and isolated NPM1-MRD evaluations

We also compared NGS-MRD and specific *NPM1*-MRD performed with the NGS error-corrected assay in the 67 patients with *NPM1* mutations. *NPM1*-MRD positivity was defined by the detection of at least one consensus read with error-corrected NGS (see methods). Forty-one patients had undetectable *NPM1*-MRD (NPM1^NEG^) including 21 NGS^NEG^ and 20 NGS^POS^. Twenty-six patients harbored detectable *NPM1-*MRD (NPM1^POS^) including 10 NGS^NEG^ and 16 NGS^POS^. All 5 patients with *NPM1* mutation over 0.1% in CR1 have at least one other mutation detected in CR1. Detectable targets both in NPM1^POS^ and NPM1^NEG^ patients were mainly *DNMT3A* and *TET2* mutations (Fig. 1, supplementary Fig. 5). NPM1^POS^ was associated with higher CIR, and lower RFS and OS probabilities (supplementary Fig. 4). We divided the patient cohort into four groups according to *NPM1-* and NGS-MRDs. CIR was significantly different between groups with particularly high risk in double positive patients (5%[0–15] vs. 19%[2–36] vs. 30%[0–60] vs. 62%[37–88] at 4 years for NPM1^NEG^NGS^NEG^, NPM1^NEG^NGS^POS^, NPM1^POS^NGS^NEG^ and NPM1^POS^NGS^POS^, respectively (*p* = 0.002). This was the same for RFS probabilities at 4 years with 90% [78–100], 71%[54–94], 50%[27–93] and 25%[11–58] respectively (*p* = 0.0004), and for OS probabilities at 4 years with 95%[86–100], 76%[60–97], 60%[36–100] and 37.5%[20–71], respectively (*p* = 0.004) (Fig. 4D–F).

We next performed a multivariable analysis (Supplementary Table 10) including *NPM1*-MRD, NGS-MRD, and ELN2017. *NPM1*- and NGS-MRD positivity were both significantly and independently associated with increased CIR (HR = 4.16[1.51–11.47] *p* = 0.0059, and HR = 3.37[1.09–10.39], *p* = 0.035, respectively). *NPM1*-MRD positivity was significantly associated with shorter EFS (HR = 3.55[1.53–8.25], *p* = 0.0032), with a similar trend for NGS-MRD positivity (HR = 2.33[0.96-5.77], *p* = 0.06). *NPM1*-MRD was the only variable associated with OS (HR = 2.97[1.15–7.67], *p* = 0.025), whereas NGS-MRD was not (HR = 2.3[0.81–6.54], *p* = 0.12).

### Evaluation of NGS-MRD and allo-SCT

Finally, we investigated whether NGS-MRD could be used to guide allo-SCT in CR1. We focused on the 127 patients with intermediate or unfavorable ELN2017, i.e. with standard allo-SCT indication. Ten patients relapsed before allo-SCT and ninety-two (72%) received allo-SCT in the first CR. The median time between allo-SCT and CR was 3.8 months [range 2.8–5.7]. Among these patients, 45 were NGS^NEG^ and 47 were NGS^POS^ (including 16 NGS ^DTA^). Considering allo-SCT as a time-dependent variable, both allo-SCT and NGS-MRD were predictive for relapse incidence and RFS. The interaction test between variables was not significant suggesting that NGS^POS^ at the time of CR does not identify a subset of patients with a specific benefit of allo-SCT. (supplementary Fig. 6 and Supplementary table 11).

## Discussion

In this study, we evaluated multi-target NGS-MRD in 189 patients treated in the ALFA-0702 study. We found that the persistence of any mutation in any gene, including DTA, was associated with unfavorable prognosis. The number of detectable gene mutations in CR1 was highly predictive of relapse and survival. NGS-MRD evaluation seems useful when compared to *NPM1*-MRD, but adds little information when compared to *WT1-*MRD in CR1. Finally, allo-SCT does not seem to abrogate the poorer prognosis associated with NGS-MRD positivity.

The value of multi-target MRD evaluation has been shown by multiple studies using stNGS [24, 25, 38] or error-corrected NGS [26, 28, 29, 32]. In these studies, the main persisting lesions were mutations found in master genes of CHIP (*DTA*) but also those detected in other epigenetic regulators (*IDH1*, *IDH2*, *BCOR)*, in splice machinery components (*SRSF2*, *U2AF1*), or in *TP53*. Overall, we observed the same mutation profile in CR in the current study.

The prognostic value of persisting DTA mutations is still debated. In one study using stNGS, *DNMT3A* mutation detection in CR had the same impact as detection of other MRD markers [38]. Conversely, in another study focused on ELN2017 intermediate patients, the detection of persistent DTA mutations in CR had a limited impact [24]. In studies using the more sensitive error-corrected NGS strategy, DTA mutations were often excluded from the analysis, considering their limited impact on CIR, although it could impact survival, potentially due to non-relapse mortality [26, 28], as patients with DTA mutations were older and treatments lead to more pronounced toxicities. In our study, the persistence of DTA mutations is associated with both RFS and OS, and there is no difference between NGS^DTA^ and NGS^other^ patients in terms of age or other disease characteristics. We hypothesize that this could be due to a more limited biological effect of the persistence of DTA mutations than the persistence of other mutations, as we observed a non-significant trend toward higher CIR in the NGS^DTA^ group in multivariable analysis. Discrepancies between studies could also be due to different inclusion or exclusion criteria. In our study, secondary and therapy-related AML cases were excluded, which could have led to an enrichment in *DNMT3A* mutated patients. Moreover, induction treatment was time-sequential and was consequently more intensive than in most studies using a 7 + 3 induction. Likewise, the various thresholds of detection of persisting mutations used across different studies could partially explain these differences. For instance, the VAF range of *TET2* mutations detected in CR is very wide, and we found only 30% of *TET2* mutations in CR in our patients with the 0.1% threshold. The number of persisting mutations could be another explanation, as only 24% of NGS^DTA^ patients have concomitant persistent mutations, in contrast to 67% in the NGS^other^ group.

Few studies have addressed the impact of the number of persistent mutations in CR. As DTA are the main mutated genes in CHIP [39], a return to the pre-leukemic clonal hematopoiesis stage could be proposed to explain the low impact of their persistence in previous studies [28]. However, the nature of the gene is not sufficient to clearly identify CHIP, which can be driven by somatic mutations in many genes [2, 39–41]. In theory, the persistence of the founding genetic event is a better way to identify the return to pre leukemic clonal hematopoiesis stage. In line with this, a previous single-center study [29] suggested that the persistence of one isolated genetic event (mutation of any gene or chromosomal event) over 0.2% after induction has little prognostic impact. In the current study, cytogenetics evaluation in CR was not performed, therefore precluding a combined cytogenetic and molecular analysis. However, the persistence of multiple mutations was clearly associated with unfavorable prognosis whereas the persistence of only one gene mutation (using the threshold of 0.1%) was not. These observations suggest that the return to pre leukemic clonal hematopoiesis defined by the persistence of only one mutation in any gene has limited prognostic value. Moreover, not all DTA mutations should be excluded from analyses, as multiple DTA mutations often co-occur [2]. It is possible that cells with persistent mutations in non-DTA genes have a higher potential of re-evolution and more frequently lead to relapse than cells with DTA mutation, as it is observed in the follow-up of individuals with CHIP [42–44].

If the prognostic value of NGS-MRD is not debated, its integration among other MRD techniques in routine clinical care remains to be assessed, due to its elevated cost and to technical difficulties in performing error-corrected NGS evaluation in patients. Multiple studies suggest an additive interest in MFC-MRD [26, 28], which was not performed in the current study. Benchmarking with other molecular markers has not been performed yet. In our study, NGS-MRD did not outperform WT1 expression monitoring in patients achieving CR or CRi. Conversely, NGS-MRD may provide additional prognostic information on top of *NPM1*-MRD, distinguishing four prognostic groups according to *NPM1-* and NGS-MRDs. This could be useful as it appears more clearly that *NPM1* mutation prognostic value is actually dependent on co-occurrent gene mutations, and probably other factors [5]. This finding has to be confirmed in larger studies, as the small number of *NPM1* mutated patients in our study limited our conclusions. Confirmation using RT-qPCR-based NPM1-MRD should also be useful, as in this study NGS NPM1 data was not compared to already published RT-PCR *NPM1*-MRD data [18], as data was not available in all patients (no material for RT-PCR at the same time point or NPM1 mutation different from type A, B or D in 12 patients).

Most previous studies suggested NGS-MRD could be useful for allo-SCT indication or for conditioning intensity decisions [24, 26, 27, 31, 38]. In our study, both NGS-MRD and allo-SCT were associated with RFS without any interaction. This suggests that the poorer prognosis associated with NGS-MRD positivity is not improved by allo-SCT and that NGS-MRD should not be factored in for transplant decisions in routine clinical practice. This was also suggested in a recent study focused on *NPM1* and *FLT3* mutations detection before allo-SCT [45]. These differences from previous studies can be due to the specificities in patient selection and to the differences in conditioning regimens [31] received between studies. These questions remain to be addressed in specific studies.

Overall, we confirmed here the high prognostic value of error-corrected multi-target NGS-MRD in a multicentric cohort of young patients with de novo AML who were homogeneously treated. DTA mutations should not be systematically excluded from MRD analysis, and the persistence of multiple markers in CR has prognostic relevance. The role of multi-target NGS as compared to other molecular MRD approaches remains to be determined, with a potentially high interest in *NPM1* mutated patients.

### Supplementary information

supplemental material

## Data Availability

All data including raw sequencing data are available on reasonable request.
