# Supplementary material for: Multi-target measurable residual disease assessed by error-corrected sequencing in patients with acute myeloid leukemia: An ALFA study
Source: Blood Cancer J. 2024 Jun 13;14(1):97. doi: 10.1038/s41408-024-01078-8 (PMC11176326; doi:10.1038/s41408-024-01078-8)
Supplement: Supplementary file 1 — supplemental material [file 41408_2024_1078_MOESM1_ESM.pdf]

Supplementary tables legends

Supplementary table 1: panel of sequenced genes at AML diagnosis

Supplementary table 2: comparison between the main characteristics of the 189 patients of the ALFA07-02 protocol included in the present study and the 387 other patients that reached CR1 in one course (without available material for MRD evaluation).

Supplementary table 3: Comparison of the outcome at 2 years of the 189 patients of the ALFA07-02 protocol included in the present study and the 387 other patients that reached CR1 in one course without available material.

Supplementary table 4: mutations identified at AML diagnosis and in first complete remission

Supplementary table 5: patients and disease characteristics

Supplementary table 6: ontogeny according to NGS status

Supplementary table 7: univariate analyses for prognosis

Supplementary table 8: number of persisting mutations in CR1 according to NGS MRD status in patients with two or more mutations at diagnosis

Supplementary table 9: multivariable analysis including WT1 and NGS MRD

Supplementary table 10: multivariable analyses including NPM1 and NGS MRD in 67 NPM1 mutated patients

Supplementary table 11: Patients outcome after allo-SCT in CR1

Supplementary figures legends

Supplementary figure 1: Comutation table at AML diagnosis and CR1.

Green boxes represent mutations detected at diagnosis and undetected in CR1. Red boxes represent mutation detected at both times. Orange boxes represent multiple mutations of the same gene

detected at AML diagnosis with at least one mutation detected in CR1. Blue boxes represent chromosomal abnormalities at AML diagnosis.

Supplementary figure 2: Comutation table in CR1 in the 100 patients with initial WT1 overexpression.

The color code is the same as supplemental figure 1. Patients with detectable NGS MRD are plotted in red in the NGS MRD line and patients without detectable mutations are plotted in green. Patients with persistent WT1 overexpression in CR1 are plotted in red in the WT1 MRD line, and patients without overexpression are plotted in light green.

Supplementary figure 3: Prognosis according to *WT1* expression in CR1 in patients with initial *WT1* over-expression.

Patients with *WT1* over-expression in CR1 are represented in yellow and patients without *WT1* over-expression in CR1 are plotted in blue.

Supplementary figure 4: Prognosis according to *NPM1* MRD by error-corrected NGS.

The threshold for positivity is defined by detection of at last one consensus read.

Supplementary figure 5: comutation table in CR1 in the 67 patients with initial *NPM1* mutation.

The color code is the same as sup figure 1. Patients with detectable NGS MRD are plotted in red in the NGS MRD line and patients without detectable mutations are plotted in green. Patients with persistent *NPM1* detection CR are plotted in red in the NPM1 MRD line, and patients without *NPM1* detection are plotted in green.

Supplementary figure 6: Interaction between allo-SCT and NGS-MRD for prognosis evaluation in the 127 intermediate or unfavorable ELN2017 patients.

RFS: Relapse free survival; OS: Overall survival. HSCT: allogeneic hematopoietic stem cell transplant.

Supplementary table 1: panel of sequenced genes at AML diagnosis

gene	NM
ASXL1	NM_015338
ASXL2	NM_018263
ATM	NM_000051
ATRX	NM_000489
BCOR	NM_001123383
BCORL1	NM_001184772
BRAF	NM_005188
CALR	NM_004343
CBL	NM_005188
CBLB	NM_001321786
CDKN2A	NM_000077
CEBPA	NM_001285829
CHEK2	NM_001005735
CEBBP	NM_001079846
CSF3R	NM_000760
CTCF	NM_001191022
CUX1	NM_001202543
DDX41	NM_001321830
DNMT3A	NM_001320892
EP300	NM_001429
ETV6	NM_001987
EZH2	NM_001203247
FBXW7	NM_001013415
FLT3	NM_004119
GATA1	NM_001145661
GATA2	NM_001145661
GNAS	NM_001077490
IDH1	NM_001282386
IDH2	NM_001289910
IKZF1	NM_001291845
JAK1	NM_001320923
JAK2	NM_001322194
JAK3	NM_000215

gene	NM
KDM6A	NM_001291415
KIT	NM_000222
KMT2C	NM_170606
KMT2D	NM_003482
KRAS	NM_004985
MPL	NM_005373
MYC	NM_002467
NF1	NM_000267
NOTCH1	NM_017617
NOTCH2	NM_001200001
NPM1	NM_002520
NRAS	NM_002524
PHF6	NM_001015877
PPM1D	NM_003620
PTEN	NM_001304717
PTPN11	NM_001330437
RAD21	NM_006265
RB1	NM_000321
RUNX1	NM_001001890
SETBP1	NM_001130110
SF3B1	NM_012433
SH2B3	NM_005475
SMC1A	NM_001281463
SMC3	NM_005445
SRSF2	NM_001195427
STAG1	NM_005862
STAG2	NM_001042749
STAT3	NM_003150
TET2	NM_001127208
TET3	NM_001287491
TP53	NM_000546
U2AF1	NM_001025203
WT1	NM_000378
ZRSR2	NM_005089

Supplementary table 2: comparison between the main characteristics of the 189 patients of the ALFA07-02 protocol included in the present study and the 387 other patients that reached CR1 in one course (without available material for MRD evaluation).

**Variable**		**Patients without material (n=387)**	**Patients included (n=189)**	**p-value**
Age (yo)		48 [38 - 54]	46 [36 - 54]	0.13
Gender n (%)	Female	183 (47.3%)	87 (46%)	0.79
	Male	204 (52.7%)	102 (54%)	
ELN2017* n (%)	Favorable	113 (31.4%)	40 (22.1%)	0.0433
	Intermediate	195 (54.2%)	105 (58%)	
	Adverse	52 (14.4%)	36 (19.9%)	
Leukocytes ($x10^9$ /L) n		7.9 [2.4 - 31.2]	7.7 [2.73 - 29.38]	0.758
Post remission treatment n (%)	HDAC	255 (65.9%)	112 (59.3%)	0.14
	CLARA	132 (34.1%)	77 (40.7%)	
ECOG performance status** n (%)	0	167 (43.5%)	80 (42.8%)	0.814
	1	179 (46.6%)	91 (48.7%)	
	2	37 (9.6%)	15 (8%)	
	3	1 (0.3%)	1 (0.5%)	

*missing in 35 **missing in 5

HDAC: High-dose Aracytine; CLARA: Clofarabine + Aracytine

Supplementary table 3: Comparison of the outcome at 2 years of the 189 patients of the ALFA07-02 protocol included in the present study and the 387 other patients that reached CR1 in one course without available material.

outcome	Patients without material (n=387)	Patients included (n=189)	p-value
2 years-PFS	0.635 [0.59-0.68]	0.622 [0.56-0.7]	0.69
2 years-OS	0.751 [0.71-0.8]	0.734 [0.67-0.8]	0.75
2 years CIR	0.277 [0.23-0.32]	0.288 [0.22-0.35]	0.57

PFS: progression free survival; OS: overall survival, CIR: cumulative incidence of relapse

Supplementary table 4: mutations identified at AML diagnosis and in first complete remission

ID	gene	CHR	Genomic location	Nucleotide change	Protein change	Sequencing depth (diag)	VAF (%) (diag)	Number of consensus read CR1	depth CR1 (consensus reads)	VAF (%) CR1
1006	NPM1	5	170837543	c.860_863dup	p.(Trp288Cysfs*?)	2080	34.2	0	11358	0
1006	RAD21	8	117875424	c.218dupA	p.(Tyr73*)	2235	39	1	13981	0.007
1006	SRSF2	17	74732959	c.284C>A	p.(Pro95His)	1202	16.7	24	12236	0.196
1006	U2AF1	21	44524456	c.104G>T	p.(Arg35Leu)	2389	27.3	8	29433	0.027
1009	EZH2	7	148504759	c.2201_2220delinsGG	p.(Leu734_Glu740delinsArg)	2047	14.2	0	19472	0
1009	NF1	17	29533315	c.1318C>T	p.(Arg440*)	2151	1.3	5	20597	0.024
1010	CEBPA	19	33792400	c.555_563dup	p.(Lys185_Arg187dup)	2966	43	0	19611	0
1010	CEBPA	19	33793119	c.303_306dup	p.(Ile103Leufs*41)	2167	39.1	0	27593	0
1010	EP300	22	41553402	c.3491G>A	p.(Cys1164Tyr)	2377	33.1	0	21266	0
1010	GATA2	3	128202768	c.952G>A	p.(Ala318Thr)	2188	8.4	1	25596	0.003
1010	TET2	4	106156072	c.973C>T	p.(Gln325*)	2895	41.7	6	21556	0.027
1010	TET2	4	106193771	c.4231G>T	p.(Glu1411*)	2844	7.2	2	27159	0.007
1011	FLT3 (ITD)	ND	ND	ND	ND	ND	ND	ND	ND	ND
1011	IDH1	2	209113112	c.395G>A	p.(Arg132His)	1341	3.9	44	28941	0.152
1011	KRAS	12	25398281	c.38G>A	p.(Gly13Asp)	1500	23.2	23	24285	0.094
1011	NRAS	1	115258744	c.38G>A	p.(Gly13Asp)	1423	3.2	3	35526	0.008
1011	NRAS	1	115258747	c.35G>A	p.(Gly12Asp)	1438	1.3	1	35757	0.002
1011	PTPN11	12	112888199	c.215C>T	p.(Ala72Val)	1859	1	8	33259	0.024
1011	WT1	11	32417918	c.1078_1082del	p.(Thr360Cysfs*6)	1180	25.3	16	31628	0.05
1013	FLT3	13	28589816	c.2564T>C	p.(Met855Thr)	1902	2.2	0	22673	0
1014	DNMT3A	2	25457242	c.2189G>A	p.(Arg730His)	1148	39.4	2163	23977	9.021
1014	DNMT3A	2	25463210	c.2283G>T	p.Met761Ile	1240	1	6	25519	0.023
1014	FLT3	13	28608262	c.1770_1793dup	p.(Tyr597_Glu598ins8)	2354	13	0	22288	0
1014	IDH1	2	209113112	c.395G>A	p.(Arg132His)	1421	10.3	4	22582	0.017
1014	NPM1	5	170837543	c.860_863dup	p.(Trp288Cysfs*?)	1471	32.1	17	18969	0.089
1014	NRAS	1	115256529	c.182A>G	p.Gln61Arg	995	0.5	1	19659	0.005
1024	DNMT3A	2	25464577	c.1481-1G>A	p.(?)	2596	44.3	3	29702	0.01
1024	FLT3 (ITD)	ND	ND	ND	ND	ND	ND	ND	ND	ND
1024	NPM1	5	170837545	c.863_864insCATG	p.(Trp288Cysfs*?)	1872	37.6	0	23344	0
1024	PTPN11	12	112926910	c.1542G>C	p.(Gln514His)	2041	1.4	0	24014	0
1024	SMC3	10	112337231	c.251A>C	p.(Asn84Thr)	1125	41.9	0	22914	0
2003	NRAS	1	115256528	c.183A>C	p.(Gln61His)	562	31.6	35	12518	0.279
2003	NRAS	1	115258748	c.34G>T	p.(Gly12Cys)	1276	9.6	4	17892	0.022
2004	KDM6A	X	44942821	c.3557T>G	p.(Leu1186Arg)	1861	16.9	2	17600	0.011
2004	RUNX1	21	36231782	c.521G>A	p.(Arg174Gln)	758	16.6	8	29433	0.027
2005	FLT3	13	28608264	c.1744_1791dup	p.(Thr582_Tyr597dup)	2692	5	0	25083	0
2005	IDH2	15	90631934	c.263G>A	p.(Arg88Gln)	2295	49.2	8	30808	0.025
2005	NPM1	5	170837544	c.863_864insCCTG	p.(Trp288Cysfs*?)	2425	41	4	23019	0.017
2006	FLT3	13	28608262	c.1770_1793dup	p.(Tyr597_Glu598ins8)	1859	12	10	25070	0.039

2006	FLT3 (ITD)	ND	ND	ND	ND	ND	ND	ND	ND	ND
2006	NRAS	1	115258747	c.35G>A	p.(Gly12Asp)	1304	3.1	56	32113	0.174
2010	CSF3R	1	36932248	c.2221C>T	p.(Gln741*)	2156	1	2	27195	0.007
2010	FLT3	13	28592642	c.2503G>T	p.(Asp835Tyr)	2462	25.7	0	25300	0
2010	KIT	4	55589766	c.1248_1255delinsCT	p.(Thr417_Asp419delinsTyr)	2899	2.4	0	22853	0
2010	KIT	4	55599321	c.2447A>T	p.Asp816Val	3799	1	2	1451	0.014
2010	RUNX1	21	36164493	c.1301A>C	p.(Asn434Thr)	2972	41.4	3656	22893	15.969
3001	FLT3	13	28592621	c.2524T>C	p.(Tyr842His)	2103	2	8	12253	0.065
3001	FLT3	13	28608263	c.1775_1792dup	p.(Val592_Tyr597dup)	2416	25.4	0	20221	0
3001	GATA2	3	128200151	c.1150_1153dup	p.(Pro385Glnfs*?)	1055	24.4	0	24825	0
3001	GATA2	3	128200690	c.1097_1114dup	p.(Gly366_Asn371dup)	1452	42	17	29955	0.056
3001	TET2	4	106196999	c.5329_5330dup	p.(His1778Phefs*43)	2358	25.7	20	20638	0.096
3001	WT1	11	32414251	c.1249C>T	p.(Arg417Cys)	1155	9	22	19564	0.112
3001	WT1	11	32417947	c.1054delinsAGA	p.(Arg353Aspfs*6)	1212	5.2	5	19062	0.026
3007	DNMT3A	2	25462022	c.2385G>T	p.Trp795Cys	413	1	7	10634	0.065
3007	DNMT3A	2	25463559	c.1666_1676del	p.(Ser556*)	211	39.8	126	13156	0.957
3007	DNMT3A	2	25470554	c.464C>T	p.(Pro155Leu)	316	43.4	129	13473	0.957
3007	GATA2	3	128202739	c.979G>T	p.Gly327Trp	342	2	2	9013	0.022
3007	KRAS	12	25398284	c.35G>A	p.Gly12Asp	444	2	0	7029	0
3007	NPM1	5	170837543	c.860_863dup	p.(Trp288Cysfs*?)	1015	37.7	1	6256	0.015
3008	ASXL1	20	31022892	c.2373_2377dup	p.(Thr793Metfs*27)	1493	42.7	605	23289	2.597
3008	IDH1	2	209113113	c.394C>T	p.(Arg132Cys)	1633	39.9	628	20699	3.033
4001	CEBPA	19	33792321	c.643G>T	p.(Glu215*)	2318	46	13	32046	0.04
4001	FLT3	13	28608262	c.1793_1794ins30	p.(Tyr597_Glu598ins10)	2358	28.5	0	21720	0
4001	KDM6A	X	44942754	c.3491_3492insAGGGTGTT	p.(Val1165Glyfs*10)	3020	1.4	0	3015	0
4001	NPM1	5	170837547	c.863_864insCAGA	p.(Trp288Cysfs*?)	1942	42.5	0	17275	0
4001	TET2	4	106156040	c.943del	p.(Ser315Profs*32)	2422	49.6	0	30823	0
4001	TET2	4	106196328	c.4664_4665del	p.(Glu1555Valfs*22)	2467	44.7	0	43209	0
4001	WT1	11	32414250	c.1250G>T	p.(Arg417Leu)	1212	45	1	23916	0.004
4004	CEBPA	19	33792378	c.550_585dup	p.(Ala184_Val195dup)	2436	36.4	0	22458	0
4004	CEBPA	19	33793186	c.76_92dup	p.(Pro32Argfs*120)	1920	35.2	0	13726	0
4004	EP300	22	41523644	c.1063del	p.(Glu355Asnfs*7)	2759	3	2	21529	0.009
4004	EP300	22	41564512	c.3934C>T	p.(Arg1312*)	2292	6.8	11	19396	0.056
4004	GATA2	3	128202759	c.961C>T	p.(Leu321Phe)	1829	30	3	18782	0.015
4004	KDM6A	X	44942730	c.3466_3467insGTTT	p.(Thr1156Serfs*3)	1321	57	0	7516	0
4005	DNMT3A	2	25457243	c.2188C>T	p.(Arg730Cys)	2653	28.9	1064	5795	18.36
4005	FBXW7	4	153244092	c.1711C>T	p.(Arg571Trp)	2493	1.2	61	6790	0.898
4005	NPM1	5	170837543	c.860_863dup	p.(Trp288Cysfs*?)	2942	20.1	0	3112	0
4005	RAD21	8	117863003	c.1474C>T	p.(Gln492*)	1791	24.1	8	3337	0.239
4007	BCOR	X	39930412	c.3052-1del	p.(?)	1181	37.2	4	20034	0.019
4007	DNMT3A	2	25457242	c.2189G>A	p.(Arg730His)	2597	41.6	8432	18262	46.172
4007	FLT3	13	28608232	c.1823_1824ins102	p.(Arg607_Glu608ins34)	3620	31.9	0	19445	0
4007	NF1	17	29556234	c.2601G>T	p.(Met867Ile)	2911	1	7	18264	0.038

4007	RUNX1	21	36171657	c.826dupT	p.(Ser276Phefs*?)	2591	29.7	0	22247	0
4010	ATRX	X	76776975	c.6977A>G	p.(Asp2326Gly)	168	2.9	1	8972	0.011
4010	CEBPA	19	33792822	c.142G>T	p.(Glu48*)	2935	4.3	2	13846	0.014
4010	DNMT3A	2	25457242	c.2189G>A	p.(Arg730His)	3399	41.1	9476	21764	43.539
4010	NPM1	5	170837545	c.863_864insCATG	p.(Trp288Cysfs*?)	2080	35.4	0	18175	0
4010	NRAS	1	115258747	c.35G>A	p.(Gly12Asp)	2372	25.5	3	25351	0.011
4010	PTPN11	12	112888301	c.317A>C	p.(Asp106Ala)	2399	2.5	6	22387	0.026
4010	PTPN11	12	112926888	c.1520G>A	p.(Gly507Glu)	2546	2.1	0	22773	0
4010	SMC3	10	112343284	c.947T>C	p.(Leu316Pro)	1064	30.2	0	18909	0
4014	NRAS	1	115258748	c.34G>T	p.(Gly12Cys)	1999	6.5	9	31337	0.028
4015	PTPN11	12	112888199	c.215C>T	p.(Ala72Val)	1530	40.1	7	32850	0.021
4015	SF3B1	2	198267484	c.1873C>T	p.(Arg625Cys)	898	3.6	200	27953	0.715
4020	U2AF1	21	44524456	c.101C>T	p.(Ser34Phe)	2741	46.9	223	19550	1.14
4021	CBL	11	119148973	c.1193A>G	p.(His398Arg)	2880	2	0	27468	0
4021	FLT3	13	28592622	c.2523C>A	p.(Asn841Lys)	3220	2.4	3	30767	0.009
4021	FLT3	13	28592640	c.2505T>G	p.(Asp835Glu)	3167	33	0	30454	0
4021	FLT3	13	28592642	c.2503G>T	p.(Asp835Tyr)	3186	8.7	1	30576	0.003
4021	IDH1	2	209113112	c.394C>G	p.(Arg132Gly)	2651	50.4	280	28583	0.979
4021	NPM1	5	170837545	c.863_864insCATG	p.(Trp288Cysfs*?)	2499	41	0	25039	0
4021	PTPN11	12	112926887	c.1519G>A	p.(Gly507Arg)	3041	1.4	0	27740	0
4022	DNMT3A	2	25457243	c.2188C>T	p.(Arg730Cys)	1505	34.2	822	22376	3.673
4022	FLT3 (ITD)	ND	ND	ND	ND	ND	ND	ND	ND	ND
4022	NPM1	5	170837543	c.860_863dup	p.(Trp288Cysfs*?)	956	28.8	100	16593	0.602
4023	GATA2	3	128200691	c.1114G>A	p.(Ala372Thr)	1737	28.5	13	29643	0.043
4023	NPM1	5	170837543	c.860_863dup	p.(Trp288Cysfs*?)	1987	39.3	0	18424	0
4023	NRAS	1	115258747	c.35G>A	p.(Gly12Asp)	2257	2	2	32805	0.006
4023	PTPN11	12	112910776	c.785T>G	p.(Leu262Arg)	1817	1.8	0	21408	0
4023	PTPN11	12	112915523	c.922A>G	p.(Asn308Asp)	2277	0.9	0	20841	0
4023	PTPN11	12	112926851	c.1483C>T	p.(Pro495Ser)	2146	5.1	9	24337	0.036
4023	RAD21	8	117869667	c.523_526dup	p.(Ala176Glufs*4)	1719	39.7	0	19678	0
4027	RUNX1	21	36171709	c.775C>T	p.(Gln259*)	1369	18.5	22	42770	0.051
4027	RUNX1	21	36171760	c.725-1G>A	p.(?)	1260	21.2	24	29157	0.082
4027	SMC3	10	112361881	c.3050T>G	p.(Leu1017Arg)	899	10.7	5	30613	0.016
4027	SRSF2	17	74732959	c.284C>A	p.(Pro95His)	815	43.8	1112	38202	2.91
4027	TET2	4	106180865	c.3894dupT	p.(Lys1299*)	686	38.9	2241	30547	7.336
4027	TET2	4	106197380	c.5713A>G	p.(Lys1905Glu)	552	31	87	40341	0.215
4029	IDH2	15	90631935	c.262C>T	p.(Arg88Trp)	2330	0.9	20	47779	0.041
4029	KRAS	12	25398281	c.38G>A	p.(Gly13Asp)	1512	11.7	5	34986	0.014
4029	NRAS	1	115258744	c.38G>A	p.(Gly13Asp)	2109	1.9	4	43093	0.009
4034	KRAS	12	25398281	c.38G>A	p.(Gly13Asp)	2093	18.5	0	22428	0
4034	NRAS	1	115256528	c.183A>C	p.(Gln61His)	1328	3.4	4	33969	0.011
4034	NRAS	1	115258746	c.35_36delinsAG	p.(Gly12Glu)	2613	3.8	0	33655	0
4034	NRAS	1	115258748	c.34G>A	p.(Gly12Ser)	2588	6	2	24924	0.008

4034	PTPN11	12	112915455	c.854T>C	p.(Phe285Ser)	2504	2.4	0	22232	0
4038	FLT3	13	28608253	c.1776_1802dup	p.(Asp593_Leu601dup)	3415	7.8	0	29707	0
4038	FLT3	13	28608253	c.1775_1831dup	p.(Val592_Leu610dup)	3415	1.3	0	29707	0
4038	KRAS	12	25378647	c.351A>T	p.(Lys117Asn)	1942	0.7	1	24131	0.004
4038	KRAS	12	25398281	c.38G>A	p.(Gly13Asp)	1587	6.9	2	26315	0.007
4038	NRAS	1	115256528	c.183A>T	p.(Gln61His)	1220	8.8	0	37505	0
4038	NRAS	1	115258744	c.38G>A	p.(Gly13Asp)	2424	9.6	2	26658	0.007
4039	ASXL1	20	31021600	c.1601_1662del	p.(Ser534Cysfs*10)	1047	4.8	0	36046	0
4039	CBL	11	119148986	c.1208_1228-47del	p.(?)	1438	4.3	0	24529	0
4039	FLT3	13	28592642	c.2503G>T	p.(Asp835Tyr)	3050	1.9	7	29247	0.023
4039	FLT3	13	28602340	c.2028C>A	p.(Asn676Lys)	2255	2.2	2	27675	0.007
5008	FLT3	13	28608250	c.1761_1805dup	p.(Asn587_Leu601dup)	4308	7.1	0	24047	0
5008	FLT3	13	28608253	c.1776_1802dup	p.(Asp593_Leu601dup)	4177	3.3	0	23589	0
5008	MYC	8	128750687	c.224C>T	p.(Pro75Leu)	4054	29.6	299	39130	0.764
5014	NPM1	5	170837545	c.863_864insTTTG	p.(Trp288Cysfs*?)	1675	36.1	35	17957	0.194
5014	NRAS	1	115258744	c.38G>T	p.(Gly13Val)	1906	16.8	35	23256	0.15
5014	PPM1D	17	58740623	c.1535del	p.(Asn512Ilefs*2)	1493	1	high noise	21732	<0.1
5014	PTPN11	12	112888165	c.181G>T	p.(Asp61Tyr)	1368	8.3	13	21644	0.06
5015	MYC	8	128750684	c.221C>T	p.(Pro74Leu)	841	48.2	13	20954	0.062
5017	CEBPA	19	33792394	c.564_569del	p.(Asn188_Glu190delinsLys)	2591	45.6	0	24034	0
5017	CEBPA	19	33793082	c.343dupG	p.(Asp115Glyfs*28)	2187	47.3	0	39920	0
5017	GATA2	3	128202759	c.961C>T	p.(Leu321Phe)	1933	42.4	7	34321	0.02
5017	NRAS	1	115258744	c.38G>A	p.(Gly13Asp)	2307	11.7	1	38447	0.002
5018	FLT3	13	28592622	c.2519_2523delinsTCCCT	p.(Ser840_Asn841delinsPhePro)	1805	24.4	6	30724	0.019
5018	SF3B1	2	198266834	c.2098A>G	p.(Lys700Glu)	2184	41.3	960	32396	2.963
5018	TET2	4	106158152	c.3054del	p.(Asn1018Lysfs*15)	2780	47.4	675	28384	2.378
5021	ASXL1	20	31022441	c.1934dupG	p.(Gly646Trpfs*12)	2319	41.4	10934	41191	26.544
5021	RUNX1	21	36164706	c.1087dupC	p.(Gln363Profs*?)	2841	1	0	31213	0
5021	RUNX1	21	36171643	c.840dupC	p.(Ser281Glnfs*?)	2396	45.3	90	32745	0.274
5021	RUNX1	21	36252865	c.416G>A	p.(Arg139Gln)	1925	40.9	113	25918	0.435
5021	TET2	4	106155941	c.845_846del	p.(Ser282*)	2116	51	6326	26816	23.59
5021	ZRSR2	X	15827428	c.544A>G	p.(Arg182Gly)	968	96.6	6637	14869	44.636
5022	FLT3	13	28608217	c.1837+1_1837+2ins114	p?	4371	13.9	0	4398	0
5022	RUNX1	21	36231782	c.521G>A	p.(Arg174Gln)	1296	41.1	73	43364	0.168
5022	RUNX1	21	36252906	c.371_375delinsGA	p.(Met124_Lys125delinsArg)	1044	35.9	9	29370	0.03
5022	SF3B1	2	198266821	c.2111T>A	p.(Ile704Asn)	1280	44.1	24	28257	0.084
5023	CTCF		67654618	c.121C>T	p.(His41Tyr)	799	33.4	4	25441	0.015
5023	DNMT3A	2	25457242	c.2188C>T	p.(Arg730Cys)	1179	32.1	917	33401	2.745
5023	FLT3	13	28592622	c.2523C>A	p.(Asn841Lys)	1132	19.8	6	31067	0.019
5023	FLT3	13	28592640	c.2505T>A	p.(Asp835Glu)	1096	4.8	0	30511	0
5023	FLT3 (ITD)	ND	ND	ND	ND	ND	ND	ND	ND	ND
5023	NPM1	5	170837543	c.860_863dup	p.(Trp288Cysfs*?)	1004	27.6	0	18441	0
5023	PTPN11	12	112888166	c.182A>G	p.(Asp61Gly)	1111	2.7	0	26295	0

5023	PTPN11	12	112888198	c.214G>A	p.(Ala72Thr)	1212	1.3	9	32380	0.027
5023	PTPN11	12	112915523	c.922A>G	p.(Asn308Asp)	1111	4.4	0	21430	0
5023	TET2	4	106196690	c.5022_5026del	p.(Ile1675Tyrfs*10)	1215	39.6	389	29916	1.3
6002	FLT3	13	28608262	c.1776_1793dup	p.(Tyr597_Glu598insAspAspPheArgGluTyr)	3144	12.6	0	14756	0
6002	GATA2	3	128200682	c.1123C>A	p.(Leu375Ile)	2269	1.5	2	12053	0.016
6002	KRAS	12	25398281	c.38G>A	p.(Gly13Asp)	1709	13.7	1	15072	0.006
6002	PTPN11	12	112888165	c.181G>T	p.(Asp61Tyr)	2420	4.7	0	17585	0
6002	PTPN11	12	112888198	c.214G>A	p.(Ala72Thr)	2529	1.1	10	14774	0.067
7003	FLT3	13	28608219	c.1836_1837ins48		3764	44	0	21471	0
7003	FLT3	13	28608284	c.1771_1772insCGGGGGGCG	p.(Tyr591delinsSerGlyGlyAsp)	2538	1.8	6	18655	0.032
7003	GATA2	3	128200720	c.1085G>A	p.(Arg362Gln)	3387	46.4	54	22411	0.24
7003	IKZF1	7	50450288	c.472G>A	p.(Gly158Ser)	2600	45	23	23219	0.099
7003	RUNX1	21	36164850	c.944T>C	p.(Ile315Thr)	4227	2.5	0	27297	0
7006	FLT3	13	28592642	c.2503G>T	p.(Asp835Tyr)	2145	45.2	6	27178	0.022
7006	NPM1	5	170837544	c.863_864insCCTG	p.(Trp288Cysfs*?)	1888	42.8	0	14497	0
7006	STAG2	X	123171474	c.385+1G>A	p.?	267	93.4	0	7708	0
7012	DNMT3A	2	25457242	c.2189G>A	p.(Arg730His)	1691	40.7	89	14275	0.623
7012	FLT3	13	28608341	c.1715A>C	p.(Tyr572Ser)	1882	9.9	0	10759	0
7012	FLT3	13	28608235	c.1722_1820dup	p.(Ser574_Pro606dup)	2297	9.8	0	15011	0
7012	FLT3	13	28608262	c.1761_1793dup	p.(Tyr597_Glu598ins11)	1963	4.5	0	14388	0
7012	IDH1	2	209113112	c.395G>A	p.(Arg132His)	1249	44.1	6	15427	0.038
7012	NPM1	5	170837543	c.860_863dup	p.(Trp288Cysfs*?)	1228	39.9	0	13308	0
7014	FLT3	13	28592642	c.2503G>T	p.(Asp835Tyr)	2245	13.2	80	14817	0.539
7014	FLT3	13	28602340	c.2028C>A	p.(Asn676Lys)	1716	19.6	1	13133	0.007
7014	PTPN11	12	112888199	c.215C>T	p.(Ala72Val)	2296	1	3	15231	0.019
7015	CEBPA	19	33792381	c.580_582dup	p.(Lys194dup)	2918	44.4	0	29717	0
7015	CEBPA	19	33792980	c.270_298del	p.(Asp91Profs*55)	2422	28.7	0	4781	0
7015	GATA2	3	128202768	c.952G>A	p.(Ala318Thr)	2607	6.3	4	30072	0.013
7016	DNMT3A	2	25457242	c.2189G>A	p.(Arg730His)	2325	44.5	72	23433	0.307
7016	FLT3	13	28592642	c.2503G>T	p.(Asp835Tyr)	2326	2.3	3	26092	0.011
7016	FLT3	13	28608229	c.1809_1826dup	p.(Glu608_Asn609insLysGluPheProArgGlu)	2832	65.8	1	23795	0.004
7016	NPM1	5	170837543	c.860_863dup	p.(Trp288Cysfs*?)	1861	37	0	21489	0
8002	PTPN11	12	112888163	c.179G>T	p.(Gly60Val)	2023	40.4	23	14822	0.155
8002	WT1	11	32413610	c.704G>C	p.(Gly235Ala)	495	40.1	38	16014	0.237
8002	WT1	11	32417942	c.469_473dup	p.(Val159Aspfs*6)	1777	1.6	1	15110	0.006
8003	NRAS	1	115258748	c.34G>A	p.(Gly12Ser)	2546	31.3	162	22400	0.723
8004	FLT3	13	28608252	c.1786_1803dup	p.(Glu596_Leu601dup)	3184	4.9	7	17838	0.039
8004	FLT3	13	28608255	c.1780_1800dup	p.(Phe594_Asp600dup)	3160	18.1	345	17734	1.945
8004	NRAS	1	115258748	c.34G>A	p.(Gly12Ser)	3145	8.8	37	26239	0.141
8004	TET2	4	106197167	c.5500C>T	p.(Gln1834*)	2905	37.6	1317	16883	7.8
8005	TP53	17	7577511	c.770T>C	p.(Leu257Pro)	1101	79.7	315	21992	1.432
8012	DNMT3A	2	25463541	c.1685C>G	p.(Ser562Cys)	1864	47	3	29755	0.01
8012	FLT3-ITD	13	28608262	c.1746_1793dup	p.(Tyr597_Glu598ins16)	2770	29.8	0	16396	0

8012	NPM1	5	170837544	c.863_864insCCTG	p.(Trp288Cysfs*?)	2713	37.4	0	14124	0
8012	NRAS	1	115258747	c.35G>A	p.(Gly12Asp)	2701	3.8	0	22344	0
8017	FLT3	13	28608217	c.1770_1837+1dup	p.(?)	3605	4.3	0	32526	0
8017	MYC	8	128750684	c.221C>A	p.(Pro74Gln)	4053	1	10	37717	0.026
8017	NRAS	1	115258747	c.35G>A	p.(Gly12Asp)	2788	4.6	4	37425	0.01
8020	FLT3	13	28592624	c.2521A>T	p.(Asn841Tyr)	709	3.5	0	24745	0
8020	FLT3	13	28608262	c.1770_1793dup	p.(Tyr597_Glu598ins8)	692	32.8	0	20539	0
8020	NPM1	5	170837543	c.860_863dup	p.(Trp288Cysfs*?)	652	35.9	0	16920	0
8020	NRAS	1	115258747	c.35G>A	p.(Gly12Asp)	717	2.5	2	26975	0.007
8020	STAG2	X	123227941	c.3654del	p.(Ile1219Leufs*15)	218	84.8	0	18463	0
8022	NPM1	5	170837545	c.863_864insCATG	p.(Trp288Cysfs*?)	1955	34.4	0	20828	0
8022	TET2	4	106156747	c.1648C>T	p.(Arg550*)	2078	13.3	0	24286	0
8022	TET2	4	106164088	c.3500+4A>G	p?	1352	38	4	18060	0.022
8022	TET2	4	106164913	c.3781C>T	p.(Arg1261Cys)	2639	10.3	11	21998	0.05
8022	TET2	4	106190797	c.4075C>T	p.(Arg1359Cys)	2189	1.1	7	20455	0.034
8022	TET2	4	106197032	c.5365_5366insAA	p.(Met1789Lysfs*32)	2384	5.8	0	22526	0
8036	IDH1	2	209113113	c.394C>T	p.(Arg132Cys)	1407	28.7	78	24919	0.313
8036	PHF6	X	133547940	c.673C>T	p.(Arg225*)	657	48.8	6	10077	0.059
8036	RUNX1	21	36164601	c.1193C>T	p.(Pro398Leu)	1655	19.3	15	17557	0.085
8036	RUNX1	21	36259175	c.231_234dup	p.(Trp79Alafs*33)	2148	10	0	20970	0
10003	SRSF2	17	74732959	c.284C>A	p.(Pro95His)	1215	1.7	15	13238	0.113
10003	WT1	11	32456559	c.332dupC	p.(Ala113Argfs*86)	1117	14.3	6	4103	0.146
10004	DNMT3A	2	25468121	c.1098+1G>A	p.(?)	2104	43.1	9870	24967	39.532
10004	IDH2	15	90631934	c.263G>A	p.(Arg88Gln)	2260	41.9	10528	27164	38.757
10005	CEBPA	19	33792369	c.568_594dup	p.(Glu190_Leu198dup)	3293	47.4	0	47802	0
10005	GATA2	3	128202759	c.961C>T	p.(Leu321Phe)	2375	3.6	5	41849	0.011
10005	GATA2	3	128202768	c.952G>A	p.(Ala318Thr)	2411	2.7	7	42811	0.016
10005	KMT2C	7	151836804	c.14416C>T	p.(Arg4806*)	2222	42	9	34318	0.026
10005	KMT2C	7	152012245	c.568C>T	p.(Arg190*)	1960	43	15	36966	0.04
11016	CBL	11	119148922	c.1142G>T	p.(Cys381Phe)	2254	36.9	16	28575	0.055
11016	FLT3	13	28608253	c.1777_1800dup	p.(Asp593_Asp600dup)	2669	35.4	3	27620	0.01
11016	SRSF2	17	74732959	c.284C>T	p.(Pro95Leu)	1990	45.3	14496	30915	46.889
11016	TET2	4	106196576	c.4910_4920del	p.(Leu1637Glnfs*20)	2412	37	15	31132	0.048
11016	TET2	4	106196705	c.5038C>T	p.(Gln1680*)	2431	56.3	31100	33840	91.903
11019	DNMT3A	2	25457242	c.2189G>A	p.(Arg730His)	2641	40	943	28579	3.299
11019	FLT3	13	28608255	c.1780_1800dup	p.(Phe594_Asp600dup)	2730	34.1	1	26156	0.003
11019	NPM1	5	170837543	c.860_863dup	p.(Trp288Cysfs*?)	2086	29.8	0	23494	0
11033	BCORL1	X	129189827	c.5076-2A>G	p.(?)	689	82.2	9	19813	0.045
11033	EZH2	7	148511220	c.1667G>A	p.(Arg556His)	1545	33.2	10	24918	0.04
11033	PHF6	X	133551319	c.955C>T	p.(Arg319*)	417	77.1	1	12269	0.008
12003	CSF3R	1	36932122	c.2346dupC	p.(Ser783Glnfs*6)	2681	15.3	15	28840	0.052
12003	FLT3	13	28608262	c.1770_1793dup	p.(Tyr597_Glu598ins8)	3616	3.7	0	21725	0
12003	NPM1	5	170837543	c.860_863dup	p.(Trp288Cysfs*?)	5242	31.5	0	18495	0

12003	PTPN11	12	112888198	c.214G>A	p.(Ala72Thr)	2897	16.1	5	26610	0.018
12003	STAG2	X	123184969	c.1018-2A>G	p.?	361	41	3	7030	0.042
12003	STAG2	X	123195620	c.1535-1G>C	p.?	283	1.6	0	8618	0
12006	ASXL1	20	31022449	c.1934del	p.(Gly645Valfs*58)	1794	38.1	high noise	25454	<0.1
12006	NF1	17	29527465	c.914_916delinsGGGT	p.(Lys305Argfs*13)	1779	42.5	0	13571	0
12006	PHF6	X	133511707	c.60_61insTC	p.(Lys21Serfs*13)	1010	96.1	0	7626	0
12010	FLT3	13	28592629	c.2516A>G	p.(Asp839Gly)	1595	2.3	0	17420	0
12010	RUNX1	21	36259171	c.239G>A	p.(Arg80His)	1740	14.5	7	16243	0.043
12010	RUNX1	21	36259214	c.187_196delinsA	p.(Val63_Asp66delinsAsn)	1893	15.4	0	17230	0
12011	FLT3	13	28608227	c.1808_1828dup	p.(Trp603_Asn609dup)	3384	2.8	0	36335	0
12011	FLT3	13	28608244	c.1770_1811dup	p.(Trp603_Glu604ins14)	3160	1.1	0	35421	0
12011	TET2	4	106157527	c.2428C>T	p.(Gln810*)	2857	38.6	50	28673	0.174
12019	KRAS	12	25398281	c.38G>A	p.(Gly13Asp)	1435	28.4	6	25040	0.023
12019	NRAS	1	115258744	c.38G>A	p.(Gly13Asp)	2310	2.6	0	33786	0
12021	FLT3	13	28592642	c.2503G>C	p.(Asp835His)	3475	31.5	0	29295	0
12021	NPM1	5	170837546	c.863_864insCCGG	p.(Trp288Cysfs*?)	1674	28.5	0	19387	0
12023	CEBPA	19	33792384	c.541_579dup	p.(Arg181_Gln193dup)	5642	49.6	0	33797	0
12023	CEBPA	19	33793002	c.276_277insTTTGACT	p.(Asp93Phefs*3)	4819	44.2	0	8773	0
12023	IDH2	15	90631934	c.263G>A	p.(Arg88Gln)	5181	2.9	9	36636	0.024
12035	CBL	11	119148966	c.1186T>C	p.(Cys396Arg)	1473	10.1	0	31360	0
12035	NPM1	5	170837543	c.860_863dup	p.(Trp288Cysfs*?)	1051	14.8	0	22111	0
12035	NRAS	1	115258747	c.35G>A	p.(Gly12Asp)	1820	4.8	1	34888	0.002
12035	STAG2	X	123185163	c.1117-2A>G	p?	817	34.5	0	9845	0
13004	FLT3	13	28608248	c.1790_1807dup	p.(Tyr597_Lys602dup)	2929	93.2	11	18448	0.059
13004	NPM1	5	170837543	c.860_863dup	p.(Trp288Cysfs*?)	3295	40.1	4	16052	0.024
13004	WT1	11	32417907	c.1090_1093dup	p.(Ala365Valfs*4)	1176	46.8	7	19579	0.035
13004	WT1	11	32417947	c.1054delinsGGG	p.(Arg352Glyfs*7)	1071	45	2	18267	0.01
13005	DNMT3A	2	25457243	c.2188C>T	p.(Arg730Cys)	1955	33.2	4768	14182	33.62
13005	KRAS	12	25380276	c.182A>C	p.(Gln61Pro)	1586	3.1	6	15224	0.039
13005	NPM1	5	170837543	c.860_863dup	p.(Trp288Cysfs*?)	2378	26.6	6	13080	0.045
13005	TET2	4	106164914	c.3782G>A	p.(Arg1261His)	2552	32.7	1244	16295	7.634
13010	ASXL2	2	25972853	c.1571del	p.(Ser524Cysfs*14)	3188	2.3	0	20729	0
13010	CEBPA	19	33792377	c.557_586dup	p.(Gln186_Val195dup)	4318	36.4	0	20146	0
13010	CEBPA	19	33793258	c.166_167dup	p.(Ser56Argfs*140)	3150	32.8	0	7414	0
13010	GATA2	3	128200720	c.1085G>A	p.(Arg362Gln)	3921	1.4	16	18436	0.086
13010	GATA2	3	128202759	c.961C>T	p.(Leu321Phe)	3399	27.6	2	17559	0.011
13011	DNMT3A	2	25457242	c.2189G>A	p.(Arg730His)	1862	44.5	29	23758	0.122
13011	EZH2	7	148523666	c.786dupC	p.(Asn263Glnfs*8)	1102	53.8	4	29014	0.013
13011	IDH2	15	90631934	c.263G>A	p.(Arg88Gln)	1581	43.2	16	29213	0.054
13011	RUNX1	21	36206802	c.629G>A	p.(Arg210Lys)	1042	85.2	3	25847	0.011
13014	GATA2	3	128202801	c.919C>T	p.(Arg307Trp)	2310	27.3	50	27897	0.179
13014	IKZF1	7	50450292	c.476A>G	p.(Asn159Ser)	1799	32	21	30585	0.068
13014	RUNX1	21	36171599	c.883_884del	p.(Ser295Asnfs*?)	2450	32.9	12	22252	0.053

13014	SF3B1	2	198266542	c.2294A>G	p.(Tyr765Cys)	1744	35.1	488	13113	3.721
13014	U2AF1	21	44514772	c.469_474dup	p.(Gln157_Tyr158dup)	1752	34.4	871	30555	2.85
13021	NPM1	5	170837543	c.860_863dup	p.(Trp288Cysfs*?)	2146	35.6	2	13345	0.014
13021	NRAS	1	115258747	c.35G>A	p.(Gly12Asp)	2096	80.1	10	20452	0.048
13021	STAG2	X	123179197	c.646C>T	p.(Arg216*)	1129	79.5	3	6777	0.044
13054	ASXL2	2	25972652	c.1772dupG	p.(Gln592Profs*16)	455	31.5	0	35488	0
13054	CEBPA	19	33792390	c.573_574insAAG	p.(Thr191_Gln192insLys)	1001	47.6	3	45633	0.006
13054	CEBPA	19	33793124	c.301_302insT	p.(Ala101Valfs*42)	768	45.9	4	35376	0.011
13054	GATA2	3	128202759	c.961C>T	p.(Leu321Phe)	783	2.8	6	37070	0.016
13054	GATA2	3	128202768	c.952G>A	p.(Ala318Thr)	803	1.6	11	38825	0.028
13054	GATA2	3	128202809	c.911C>A	p.(Pro304His)	803	11.1	6	37070	0.016
13054	RAD21	8	117864787	c.1321+1G>C	p?	384	12.5	2	26380	0.007
13064	FLT3	13	28608216	c.1786_1837+2dup	p?	2496	33.3	0	22129	0
13064	NPM1	5	170837548	c.864_865delinsCCTCAA	p.(Trp288Cysfs*?)	3061	43.7	0	19965	0
14002	NPM1	5	170837543	c.860_863dup	p.(Trp288Cysfs*?)	5039	32.1	4	14950	0.026
14002	NRAS	1	115258747	c.35G>A	p.(Gly12Asp)	3951	3	0	23181	0
14002	PTPN11	12	112888199	c.215C>A	p.(Ala72Asp)	3307	3.6	6	20707	0.028
14002	TET2	4	106196893	c.5228dupA	p.(Asn1743Lysfs*10)	3808	42.6	4	17215	0.023
14005	WT1	11	32456321	c.570del	p.(Gly191Alafs*95)	1257	39.4	8	19843	0.04
15002	FLT3	13	28608257	c.1798_1799ins36	p.(Tyr599_Asp600ins12)	4256	44	0	19201	0
15002	NPM1	5	170837543	c.860_863dup	p.(Trp288Cysfs*?)	5190	34.9	3	17199	0.017
15002	PHF6	X	133511716	c.73_74del	p.(Asp25Glnfs*10)	1528	1.9	0	9408	0
15005	BRAF	7	140453136	c.1799T>A	p.(Val600Glu)	1955	1.8	6	19238	0.031
15005	DNMT3A	2	25467448	c.1172G>A	p.(Gly391Asp)	2192	41.6	14	37437	0.037
15005	MYC	8	128750683	c.220C>T	p.(Pro74Ser)	2905	44.7	8	31809	0.025
15005	NPM1	5	170837544	c.863_864insCCTG	p.(Trp288Cysfs*?)	2466	39.6	1	15480	0.006
15005	NRAS	1	115258744	c.38G>A	p.(Gly13Asp)	2185	21.7	2	28631	0.006
15005	NRAS	1	115258747	c.35G>A	p.(Gly12Asp)	2203	1.7	3	28904	0.01
15005	PTPN11	12	112888199	c.215C>G	p.(Ala72Gly)	1979	3	4	27253	0.014
15005	PTPN11	12	112926884	c.1516T>C	p.(Ser506Pro)	2179	2.7	1	25497	0.003
15005	RAD21	8	117864787	c.1321+1G>A	p.(?)	1448	42.9	7	20524	0.034
15012	RUNX1	21	36171724	c.759del	p.(Tyr254Thrfs*30)	2597	44.4	18	25626	0.07
15012	RUNX1	21	36252959	c.322G>A	p.(Gly108Ser)	2756	43.9	19	26435	0.071
15012	SF3B1	2	198266833	c.2099delinsTTCT	p.(Lys700delinsIleLeu)	2356	40.4	14	27106	0.051
15016	ASXL1	20	31022449	c.1934del	p.(Gly645Valfs*58)	2066	12.2	high noise	23133	<0.1
15016	RUNX1	21	36231782	c.521G>A	p.(Arg174Gln)	1332	10.2	5	26323	0.018
15016	RUNX1	21	36252866	c.414_415insAAAGGGGGAG	p.(Gly138_Arg139insLysGlyGlyGly)	2438	11.8	0	19684	0
16007	DNMT3A	2	25469085	c.916del	p.(Arg306Glyfs*193)	4314	46.7	989	23928	4.133
16007	IDH1	2	209113112	c.395G>A	p.(Arg132His)	2946	10.9	11	22553	0.048
16007	NPM1	5	170837543	c.860_863dup	p.(Trp288Cysfs*?)	3409	31.1	18	16360	0.11
16007	NRAS	1	115258747	c.35G>A	p.(Gly12Asp)	3683	22.3	4	28212	0.014
16007	PTPN11	12	112926852	c.1484C>T	p.(Pro495Leu)	3508	0.8	2	19974	0.01
16007	WT1	11	32417907	c.505_508dup	p.(Ala170Valfs*4)	2294	4.5	17	22477	0.075

16013	ASXL1	20	31022549	c.2034G>T	p.(Arg678Ser)	2111	1	69	27175	0.253
16013	DNMT3A	2	25457243	c.2188C>T	p.(Arg730Cys)	2983	46.8	14	29568	0.047
16013	FLT3	13	28602376	c.1992G>A	p.(Met664Ile)	2699	3.3	7	25031	0.027
16013	FLT3	13	28608262	c.1770_1793dup	p.(Tyr597_Glu598ins8)	4035	9.7	0	24481	0
16013	NPM1	5	170837543	c.860_863dup	p.(Trp288Cysfs*?)	3660	38.7	0	20278	0
16013	NRAS	1	115258747	c.35G>T	p.(Gly12Val)	3291	6.1	3	34610	0.008
16013	NRAS	1	115258747	c.35G>A	p.(Gly12Asp)	2228	4.9	0	34610	0
16013	PTPN11	12	112888198	c.214G>A	p.(Ala72Thr)	2975	24.9	3	30189	0.009
16013	SMC1A	X	53438772	c.1127G>A	p.(Arg376Gln)	1042	49.4	13	17176	0.075
16013	SRSF2	17	74732959	c.284C>A	p.(Pro95His)	1643	1.2	50	26855	0.186
16016	NRAS	1	115256535	c.176C>A	p.(Ala59Asp)	842	3.9	2	5661	0.035
16016	TET2	4	106196312	c.4645C>A	p.(Pro1549Thr)	1634	37.5	5	15021	0.033
16017	WT1	11	32414302	c.1199-1G>C	p?	1305	17.5	545	17795	3.062
16017	WT1	11	32417871	c.1138_1142dupCGGTC	p.Ala382Glyfs*69	1517	0.5	1	23649	0.004
16017	WT1	11	32456332	c.559del	p.(Gln187Argfs*99)	1327	2	11	23776	0.046
16019	KMT2D	12	49445274	c.2191del	p.(Glu731Serfs*199)	1231	18	0	28997	0
16019	NF1	17	29553477	c.2033dupC	p.(Ile679Aspfs*21)	841	4.9	16	19963	0.08
16019	NF1	17	29661945	c.5839C>T	p.(Arg1947*)	1530	4.9	14	18636	0.075
16019	TP53	17	7576924	c.920-15_921del	p.(?)	899	70	11218	26907	41.691
16021	IDH2	15	90631838	c.359G>A	p.(Arg120Lys)	1454	36.8	6	18276	0.032
16021	WT1	11	32414218	c.1282C>T	p.(His428Tyr)	1081	36.5	1	14264	0.007
16023	FLT3	13	28592640	c.2505T>A	p.(Asp835Glu)	2437	1	3	26607	0.011
16023	FLT3	13	28592642	c.2503G>T	p.(Asp835Tyr)	2437	3.5	1	26724	0.003
16023	NPM1	5	170837547	c.863_864insTCGG	p.(Trp288Cysfs*?)	2724	9.2	0	18931	0
16023	PTPN11	12	112888197	c.213T>A	p.(Phe71Leu)	1948	5	1	26698	0.003
16029	DNMT3A	2	25463170	c.1866+1G>A	p.(?)	2224	44.9	2313	26060	8.875
16029	DNMT3A	2	25464525	c.1532C>T	p.(Ser511Leu)	2485	44	3309	39497	8.377
16029	NPM1	5	170837543	c.860_863dup	p.(Trp288Cysfs*?)	2463	35.8	79	26534	0.297
16029	NRAS	1	115256529	c.182A>G	p.(Gln61Arg)	987	15.1	2	28864	0.006
16029	NRAS	1	115258747	c.35G>A	p.(Gly12Asp)	2145	0.5	1	39370	0.002
16029	NRAS	1	115258748	c.34G>A	p.(Gly12Ser)	2138	1.1	5	39450	0.012
16029	PTPN11	12	112888211	c.227A>G	p.(Glu76Gly)	2041	2.8	69	37502	0.183
16037	ASXL2	2	25972864	c.1560_1571delinsCGGAAATAGGACT	p.(Leu521Glyfs*?)	1888	6.1	0	32138	0
16037	CEBPA	19	33792603	c.354_360dup	p.(Ala121Argfs*83)	1122	37	4589	15971	28.733
16037	CEBPA	19	33793029	c.249dupC	p.(Thr84Hisfs*10)	1822	41.8	0	16890	0
16037	IKZF1	7	50450284	c.468G>C	p.(Gln156His)	1478	30.6	3	39746	0.007
16037	KMT2C	7	151849833	c.12482_12483insGGGACTAA	p.(Tyr4161*)	2179	7.9	0	26350	0
16037	RAD21	8	117878960	c.9C>A	p.(Tyr3*)	1637	44	2	11358	0.017
16038	WT1	11	32413556	c.1343A>G	p.(His448Arg)	318	3.3	0	22693	0
16040	ASXL2	2	25966418	c.2788G>T	p.(Gly930*)	2548	1.3	4	39593	0.01
16040	CEBPA	19	33792361	c.567_602dup	p.(Ser200_Asp201ins12)	3483	46.7	0	69913	0
16040	CEBPA	19	33793252	c.26del	p.(Pro9Argfs*137)	2839	53.5	10	27936	0.035
16040	GATA2	3	128200720	c.1085G>A	p.(Arg362Gln)	2638	46.5	27	55452	0.048

**16040**	KMT2C	7	151874001	c.8535_8536dup	p.(Lys2846Ilefs*18)	1553	88.2	0	29824	**0**
16041	FLT3	13	28592642	c.2503G>T	p.(Asp835Tyr)	2250	27.5	1	27087	**0.003**
**16041**	NPM1	5	170837543	c.860_863dup	p.(Trp288Cysfs*?)	1757	26	0	19621	**0**
**16041**	STAG2	X	123224611	c.3467+4_3467+7del	p?	941	57.3	1	8316	**0.012**
16043	CEBPA	19	33792400	c.558_563dup	p.(Arg187_Asn188insLysArg)	2000	46.6	0	24211	**0**
**16043**	CEBPA	19	33793119	c.156_159dup	p.(Ile54Leufs*41)	1701	42.4	0	15931	**0**
**16043**	GATA2	3	128202767	c.953C>T	p.(Ala318Val)	1564	42.7	1	20169	**0.004**
16044	FLT3	13	28592642	c.2503G>A	p.(Asp835Asn)	1772	1.6	7	35416	**0.019**
16046	DNMT3A	2	25457242	c.2189G>A	p.(Arg730His)	2150	45	331	26120	**1.267**
**16046**	FLT3	13	28592642	c.2503G>T	p.(Asp835Tyr)	2162	5	2	24995	**0.008**
**16046**	FLT3	13	28608225	c.1830_1831ins78	p.(Leu610_Glu611ins26)	3127	57.2	0	20538	**0**
**16046**	NPM1	5	170837543	c.860_863dup	p.(Trp288Cysfs*?)	3304	38	0	15099	**0**
**16046**	NRAS	1	115258747	c.35G>A	p.(Gly12Asp)	2380	1.8	2	28048	**0.007**
16050	EZH2	7	148506443	c.2054G>A	p.(Arg685His)	2440	10.5	0	19333	**0**
**16050**	NPM1	5	170837543	c.860_863dup	p.(Trp288Cysfs*?)	2321	35.6	0	26588	**0**
**16050**	SMC3	10	112359463	c.2320A>T	p.(Arg774*)	1728	9.7	2	27685	**0.007**
**16050**	TET2	4	106158409	c.3310_3311insA	p.(Phe1104Tyrfs*26)	2943	39.5	7	33230	**0.021**
**16050**	TET2	4	106190775	c.4053T>A	p.(Tyr1351*)	2396	6	1	24828	**0.004**
**16050**	TET2	4	106193849	c.4317dupA	p.(Arg1440Thrfs*38)	2959	19.5	2	29934	**0.006**
17005	TP53	17	7579358	c.329G>C	p.(Arg110Pro)	2511	41.4	34	31276	**0.108**
17012	DNMT3A	2	25457243	c.2188C>T	p.(Arg730Cys)	1813	39.8	5949	26927	**22.093**
**17012**	GATA2	3	128200709	c.1095_1096insCAC	p.(Asn365_Gly366insHis)	2046	45.7	0	21676	**0**
**17012**	KIT	4	55599321	c.2447A>T	p.(Asp816Val)	728	43.2	3	21900	**0.013**
**17012**	NPM1	5	170837543	c.860_863dup	p.(Trp288Cysfs*?)	1395	42.8	0	24740	**0**
17014	TP53	17	7578211	c.638G>T	p.(Arg213Leu)	2956	14.7	7233	34628	**20.887**
18006	CEBPA	19	33792381	c.580_582dup	p.(Lys194dup)	919	46.1	0	35477	**0**
**18006**	CEBPA	19	33793018	c.392_407del	p.(Gly131Alafs*59)	721	57.7	0	10161	**0**
**18006**	GATA2	3	128202767	c.953C>T	p.(Ala318Val)	694	49.1	12	31107	**0.038**
**18006**	KRAS	12	25378647	c.351A>T	p.(Lys117Asn)	686	5.8	1	22106	**0.004**
**18006**	MYC	8	128750684	c.221C>T	p.(Pro74Leu)	812	29.1	8	32508	**0.024**
20002	BCOR	X	39914639	c.4620del	p.(Met1541Trpfs*5)	685	3.7	2169	7359	**29.474**
**20002**	BCOR	X	39916538	c.4362dupT	p.(Asp1455*)	940	20.2	214	8573	**2.496**
**20002**	BCOR	X	39932952	c.1647delinsGCGGT	p.(Thr550Argfs*8)	1089	19.6	943	9314	**10.124**
**20002**	NPM1	5	170837544	c.863_864insCCTG	p.(Trp288Cysfs*?)	3349	7	0	11586	**0**
**20002**	TET2	4	106197258	c.5591T>A	p.(Val1864Glu)	2057	6	1	18369	**0.005**
20010	DNMT3A	2	25466800	c.1447C>T	p.(Arg483Trp)	1754	31	5050	24094	**20.959**
**20010**	FBXW7	4	153244137	c.1666C>T	p.(Arg556Trp)	1841	21.9	45	25178	**0.178**
**20010**	FLT3	13	28602340	c.2028C>G	p.(Asn676Lys)	1939	21.7	0	21034	**0**
**20010**	NPM1	5	170837547	c.863_864insTATG	p.(Trp288Cysfs*?)	2482	24.4	0	15402	**0**
**20010**	TET2	4	106158283	c.3188_3189del	p.(Thr1063Serfs*4)	3310	27.5	0	21732	**0**
22003	BCOR	X	39930414	c.3052-2A>G	p.(?)	1927	19	7	23506	**0.029**
**22003**	BCORL1	X	129190010	c.5264dupC	p.(Gly1756Argfs*4)	3996	16.1	29	31647	**0.09**
**22003**	ETV6	12	12037507	c.1138T>G	p.(Trp380Gly)	2521	14.7	2	26509	**0.007**

22003	RUNX1	21	36253009	c.271dupG	p.(Val91Glyfs*20)	3917	15.8	0	19931	0
22003	WT1	11	32414284	c.1216T>C	p.(Cys406Arg)	2122	2	0	23316	0
22003	WT1	11	32417907	c.505_508dup	p.(Ala170Valfs*4)	2594	3.4	0	25723	0
22004	FLT3	13	28608277	c.1731_1778dup	p.(Val592_Asp593ins16)	3942	7.3	0	28507	0
22004	WT1	11	32413578	c.736C>T	p.(Arg246*)	678	11.7	152	30493	0.498
22004	WT1	11	32417802	c.613+1G>C	p.(?)	2572	1.7	130	24593	0.528
22004	WT1	11	32417907	c.505_508dup	p.(Ala170Valfs*4)	2616	24.8	216	30362	0.711
22007	DNMT3A	2	25457243	c.2188C>T	p.(Arg730Cys)	788	42	152	17748	0.856
22007	IDH2	15	90631934	c.263G>A	p.(Arg88Gln)	1145	48.5	205	24501	0.836
22007	KDM6A	X	44929228	c.2484C>A	p.(Asp828Glu)	1443	1.3	4	11741	0.034
22007	SRSF2	17	74732959	c.284C>A	p.(Pro95His)	830	41.2	236	14451	1.633
22009	CEBPA	19	33792384	c.577_579dup	p.(Gln193dup)	2061	48	35	44461	0.078
22009	CEBPA	19	33793147	c.131_132insTCTGCGAGATCTG	p.(Glu45Leufs*53)	1492	49.8	384	40188	0.955
22009	FLT3 (ITD)	ND	ND	ND	ND	ND	ND	ND	ND	ND
22009	NRAS	1	115258748	c.34G>A	p.(Gly12Ser)	2107	31.8	11	30394	0.036
22009	PTPN11	12	112888198	c.214_215delinsTT	p.(Ala72Phe)	1918	2.2	4	27319	0.014
22009	RAD21	8	117870603	c.469G>T	p.(Glu157*)	1029	40.2	13	8305	0.156
23001	DNMT3A	2	25457242	c.2189G>A	p.(Arg730His)	2258	40	69	24451	0.282
23001	FLT3	13	28608286	c.1734_1769dup	p.(Tyr589_Phe590ins12)	2696	43.9	1	20899	0.004
23001	NPM1	5	170837543	c.860_863dup	p.(Trp288Cysfs*?)	2020	36.3	1	18678	0.005
23001	NRAS	1	115258745	c.37G>T	p.(Gly13Cys)	2192	7.4	5	28293	0.017
23001	NRAS	1	115258748	c.34G>A	p.(Gly12Ser)	2191	8	7	28598	0.024
23001	TET2	4	106197444	c.5777G>C	p.(Arg1926Pro)	3232	1	5	23969	0.02
23004	CHEK2	22	29092947	c.1166G>A	p.(Arg389His)	1587	36	594	15190	3.91
23004	JAK2	9	5073770	c.1849G>T	p.(Val617Phe)	2534	54.7	582	12417	4.687
23004	RUNX1	21	36231792	c.511C>A	p.Asp171Asn	1708	35.6	3	23104	0.012
23008	DNMT3A	2	25470546	c.472A>T	p.(Ile158Phe)	2380	29	3349	30240	11.074
23008	EZH2	7	148506443	c.2054G>A	p.(Arg685His)	1838	8.4	4	18233	0.021
23008	EZH2	7	148511105	c.1782G>A	p.(Trp594*)	1778	3.3	6	24256	0.024
23008	IDH2	15	90631838	c.359G>A	p.(Arg120Lys)	2673	9.7	5	32126	0.015
23012	CEBPA	19	33792455	c.509G>A	p.(Arg170His)	3107	36.9	5	17118	0.029
23012	ETV6	12	11992093	c.183G>A	p.(Trp61*)	2464	41.7	11	19018	0.057
23012	ETV6	12	11992223	c.314_315insGGG	p.(Arg105_Ser106insGly)	3262	39.4	0	19999	0
23012	WT1	11	32456467	c.424dupC	p.(Gln142Profs*57)	2610	36	high noise	high noise	<0.1
23013	FLT3	13	28608232	c.1823_1824ins72	p.(Arg607_Glu608ins24)	671	30.5	0	27909	0
23013	NPM1	5	170837543	c.860_863dup	p.(Trp288Cysfs*?)	415	33.9	1	21783	0.004
23013	TET2	4	106157390	c.2290dupC	p.(Gln764Profs*5)	540	37.7	224	33358	0.671
23013	TET2	4	106180888	c.3915_3921del	p.(Pro1306Serfs*55)	371	47	793	26826	2.956
23015	DNMT3A	2	25457242	c.2189G>A	p.(Arg730His)	1987	44.5	5928	22687	26.129
23015	IDH1	2	209113112	c.395G>A	p.(Arg132His)	1843	1.2	3	25589	0.011
23015	NPM1	5	170837543	c.860_863dup	p.(Trp288Cysfs*?)	2336	37.6	3	22349	0.013
23016	CBL	11	119148759	c.1096-114_1431+2818del	p.(?)	1428	4.9	0	26733	0
23016	CSF3R	1	36932224	c.2245C>T	p.(Gln749*)	2137	3	7	31836	0.021

23016	KRAS	12	25398284	c.35G>A	p.(Gly12Asp)	1422	18.1	0	29333	0
23016	NRAS	1	115258744	c.38G>A	p.(Gly13Asp)	2098	1.3	0	34525	0
23016	PTPN11	12	112926885	c.1517C>T	p.(Ser506Leu)	2133	3.5	1	31105	0.003
23016	TP53	17	7574003	c.1024C>T	p.(Arg342*)	1779	60.8	110	32673	0.336
23023	FBXW7	4	153244091	c.1712G>A	p.(Arg571Gln)	1359	79.7	10	19433	0.051
23023	FLT3	13	28592642	c.2503G>T	p.(Asp835Tyr)	1554	35.3	2	27619	0.007
23023	NPM1	5	170837547	c.863_864insCTTG	p.(Trp288Cysfs*?)	1448	36.2	0	20828	0
23023	NRAS	1	115258748	c.34G>T	p.(Gly12Cys)	1600	1	0	29773	0
23023	STAG2	X	123202412	c.2266-2A>G	p?	348	84.2	0	7184	0
23026	NF1	17	29548948	c.1721+1G>A	p?	851	45.4	8	15972	0.05
23026	NRAS	1	115258747	c.35G>A	p.(Gly12Asp)	2064	1	2	37057	0.005
23026	PTPN11	12	112926885	c.1517C>T	p.(Ser506Leu)	2085	34.5	4	33788	0.011
23026	TET2	4	106193901	c.4363A>G	p.(Arg1455Gly)	2496	37.1	1	31435	0.003
23027	DNMT3A	2	25457242	c.2189G>A	p.(Arg730His)	2649	42.2	4320	38580	11.197
23027	FLT3	13	28608218	c.1837+1_1837ins63	p.(?)	3816	38.3	0	36090	0
23027	KRAS	12	25398281	c.38G>A	p.(Gly13Asp)	2017	1.4	5	32300	0.015
23027	NPM1	5	170837546	c.863_864insCCAG	p.(Trp288Cysfs*?)	3379	41.3	1	29617	0.003
23027	NRAS	1	115258747	c.35G>A	p.(Gly12Asp)	2876	2.6	1	41568	0.002
23027	PTPN11	12	112926888	c.1520G>T	p.(Gly507Val)	2961	6.8	2	36258	0.005
23027	SMC1A	X	53432720	c.1648C>T	p.(Pro550Ser)	2005	43.6	6	43268	0.013
23029	BCOR	X	39922189	c.3880del	p.(Gln1294Argfs*41)	3508	35.4	21	20847	0.1
23029	CBL	11	119144603	c.616C>T	p.(Arg206*)	1198	95.4	1059	24496	4.323
23029	IKZF1	7	50450292	c.476A>G	p.(Asn159Ser)	2424	13.1	182	26620	0.683
23029	PHF6	X	133551265	c.902dupA	p.(Tyr301*)	1524	47.1	9272	25501	36.359
23029	PTPN11	12	112915523	c.922A>G	p.(Asn308Asp)	2382	1.5	0	23319	0
23029	TET2	4	106164914	c.3782G>A	p.(Arg1261His)	2952	27.3	0	27097	0
23029	TET2	4	106197285	c.5618T>C	p.(Ile1873Thr)	2830	26.7	14	26980	0.051
23029	U2AF1	21	44524456	c.101C>T	p.(Ser34Phe)	2612	45.4	285	29058	0.98
24015	TP53	17	7578247	c.601_612delinsACGCGG	p.Leu201_Glu204delinsGlyAsnProArg	1678	82.5	0	31942	0
24021	CEBPA	19	33792710	c.238_253del	p.(Ala80Argfs*114)	844	41.5	0	20643	0
24021	CEBPA	19	33792986	c.438_439dup	p.(Pro147Argfs*49)	590	39.1	0	10538	0
24021	GATA2	3	128202758	c.962T>A	p.(Leu321His)	1054	49.8	0	25130	0
24021	IDH2	15	90631934	c.263G>A	p.(Arg88Gln)	1137	49.6	2	29105	0.006
24021	IKZF1	7	50450288	c.472G>A	p.(Gly158Ser)	817	43	1	26533	0.003
24021	KDM6A	X	44942816	c.3552_3553insAGGC	p.(Gln1185Argfs*19)	583	40.4	0	19577	0
24021	NRAS	1	115258747	c.35G>C	p.(Gly12Ala)	828	47.7	0	26968	0
24025	FLT3 (ITD)	ND	ND	ND	ND	ND	ND	ND	ND	ND
24025	IDH1	2	209113113	c.394C>T	p.(Arg132Cys)	2352	42.8	100	13763	0.726
24025	NPM1	5	170837543	c.860_863dup	p.(Trp288Cysfs*?)	2894	34.3	0	7702	0
24027	ETV6	12	12022644	c.751_755dup	p.(Lys254Thrfs*14)	2619	10.7	0	40894	0
24027	IDH1	2	209113112	c.395G>A	p.(Arg132His)	1767	2.2	5	31299	0.015
24027	TET2	4	106164061	c.3571C>T	p.(Gln1191*)	2016	10.4	1	32768	0.003
24027	WT1	11	32450089	c.86del	p.(Asn29Thrfs*45)	1390	40.9	0	31036	0

24029	KRAS	12	25398284	c.35G>T	p.(Gly12Val)	874	27.7	15	30060	0.049
24029	KRAS	12	25398285	c.34G>T	p.(Gly12Cys)	874	7.5	11	30003	0.036
24032	FLT3	13	28592629	c.2516A>G	p.(Asp839Gly)	2850	38.1	2	32477	0.006
24032	IDH1	2	209113112	c.395G>A	p.(Arg132His)	1901	39.8	20	27726	0.072
24032	MYC	8	128750683	c.220C>T	p.(Pro74Ser)	2722	42.8	23	30374	0.075
24032	NRAS	1	115258747	c.35G>A	p.(Gly12Asp)	2422	3.3	3	29032	0.01
24033	BCOR	X	39922236	c.3832_3833del	p.(Ala1278Thrfs*13)	631	80.1	39	10329	0.377
24033	BCORL1	X	129190010	c.5264dupC	p.(Gly1756Argfs*4)	960	32.4	13	24265	0.05
24033	CBL	11	119148875	c.1096-1G>T	p?	1139	21.1	1	19209	0.005
24033	CUX1	7	101758554	c.707+1G>A	p?	645	78.2	3166	28324	11.177
24033	EP300	22	41560068	c.3740G>A	p.(Cys1247Tyr)	1561	17.3	51	29739	0.171
24033	KRAS	12	25398285	c.34G>C	p.(Gly12Arg)	1157	15	30	24461	0.122
24033	SRSF2	17	74732959	c.284C>A	p.(Pro95His)	1773	37.8	2345	33487	7.002
24033	STAG2	X	123171416	c.328C>T	p.(Arg110*)	288	27.1	27	10349	0.26
24033	STAG2	X	123217308	c.2962C>T	p.(Gln988*)	420	40.8	2	12599	0.015
24033	TET2	4	106156707	c.1605_1608del	p.(Asn535Lysfs*6)	1600	40	1916	35274	5.431
24036	CBL	11	119148966	c.1186T>C	p.(Cys396Arg)	2390	1.6	0	17939	0
24036	FLT3	13	28592629	c.2516A>G	p.(Asp839Gly)	4585	1.8	0	23320	0
24036	FLT3	13	28592640	c.2505T>A	p.(Asp835Glu)	4445	3.6	2	22637	0.008
24036	FLT3	13	28592642	c.2503G>T	p.(Asp835Tyr)	4423	21.7	5	22951	0.021
24036	NPM1	5	170837546	c.863_864insTTCG	p.(Trp288Cysfs*?)	2932	31.1	0	15493	0
24036	STAG2	X	123217389	c.3043A>T	p.(Lys1015*)	814	68.7	0	8100	0
24036	ZRSR2	X	15827401	c.517_518insGG	p.(Pro173Argfs*66)	1895	67.3	0	11875	0
24037	FLT3	13	28592641	c.2504A>C	p.(Asp835Ala)	2474	34.6	0	27779	0
24037	KRAS	12	25398281	c.38G>A	p.(Gly13Asp)	1631	2.5	5	25912	0.019
24038	ATRX	X	76939939	c.809C>T	p.(Pro270Leu)	1527	13.7	2	17127	0.011
24038	IDH2	15	90631838	c.359G>A	p.(Arg120Lys)	1962	20.3	1	18915	0.005
24038	PTPN11	12	112926851	c.1483C>T	p.(Pro495Ser)	2080	13.8	0	15806	0
24040	ASXL1	20	31022287	c.1772dupA	p.(Tyr591*)	2647	19.6	7	28131	0.024
24040	BCOR	X	39933926	c.673C>T	p.(Gln225*)	3771	23.4	17	25593	0.066
24040	BCORL1	X	129149098	c.2350C>T	p.(Arg784*)	3701	15.2	10	30169	0.033
24040	CSF3R	1	36932224	c.2245C>T	p.(Gln749*)	2652	3.4	4	35068	0.011
24040	RUNX1	21	36259173	c.237G>T	p.(Trp79Cys)	3040	42.2	16	30364	0.052
24040	WT1	11	32417900	c.1093_1100 delinsCCCTTAAT	p.Ala367Profs*?	2159	1.7	0	27043	0
24042	DNMT3A	2	25457242	c.2189G>A	p.(Arg730His)	1314	42.1	3759	17092	21.992
24042	FLT3	13	28608231	c.1795_1824dup	p.(Tyr599_Glu608dup)	1886	90.9	0	17728	0
24042	FLT3 (ITD)	ND	ND	ND	ND	ND	ND	ND	ND	ND
24042	NPM1	5	170837543	c.860_863dup	p.(Trp288Cysfs*?)	1092	38	2	15752	0.012
24043	BCOR	X	39930888	c.3051+2T>G	p?	860	46.7	1	30048	0.003
24043	KRAS	12	25378562	c.436G>A	p.(Ala146Thr)	1455	35	4	32714	0.012
24044	FLT3	13	28592642	c.2503G>T	p.(Asp835Tyr)	3644	35.4	1	21947	0.004
24044	IDH2	15	90631934	c.263G>A	p.(Arg88Gln)	3908	37.6	10	23368	0.042
24044	NPM1	5	170837547	c.863_864insTATG	p.(Trp288Cysfs*?)	3115	32.5	0	16747	0

25002	FLT3	13	28608231	c.1824_1825ins60	p.(Glu608_Asn609ins20)	3663	1.6	0	18329	0
25002	MYC	8	128750695	c.232C>A	p.Pro78Thr	3155	5.9	high noise	high noise	<0.1
25007	RUNX1	21	36252941	c.340T>C	p.(Ser114Pro)	2998	31.3	4	26383	0.015
25007	RUNX1	21	36259145	c.255_264del	p.(Pro86Serfs*6)	2949	30.9	0	23974	0
25007	SF3B1	2	198266834	c.2098A>G	p.(Lys700Glu)	3211	31.5	0	28842	0
25025	CEBPA	19	33792382	c.581_582insCCA	p.(Lys194delinsAsnGln)	1999	46.7	0	12586	0
25025	CEBPA	19	33793029	c.396dupC	p.(Thr133Hisfs*10)	1069	43.2	0	35182	0
25025	DNMT3A	2	25466763	c.1480+4A>T	p.?	731	39.7	78	34225	0.227
25025	MYC	8	128750683	c.220C>G	p.(Pro74Ala)	1988	47.8	0	30036	0
25025	RAD21	8	117864185	c.1470+2delinsGG	p?	1527	44.7	1	25160	0.003
26005	CEBPA	19	33792381	c.580_582dup	p.(Lys194dup)	3458	45.3	20	33289	0.06
26005	CEBPA	19	33793082	c.343_344insGTCG	p.(Asp115Glyfs*29)	2638	45	16	19565	0.081
26005	CSF3R	1	36933434	c.1853C>T	p.(Thr618Ile)	1846	33.4	21	32498	0.064
26007	DNMT3A	2	25467449	c.1171G>T	p.(Gly391Cys)	2890	43.6	1112	39321	2.828
26007	FLT3	13	28608260	c.1795_1796ins60	p.(Glu598_Tyr599ins20)	3747	8	0	14271	0
26007	NPM1	5	170837543	c.860_863dup	p.(Trp288Cysfs*?)	2149	38.2	0	11992	0
26009	ASXL1	20	31022592	c.2077C>T	p.(Arg693*)	593	4.1	29	28132	0.103
26009	CUX1	7	101844765	c.2221C>T	p.(Gln741*)	594	3.1	2	35404	0.005
26009	DDX41	5	176939370	c.1196G>A	p.(Arg399His)	581	3.8	13	31549	0.041
26010	CEBPA	19	33793146	c.280G>T	p.(Glu94*)	2858	49.1	9	26999	0.033
26010	FLT3	13	28608248	c.1790_1807dup	p.(Tyr597_Lys602dup)	2940	43.4	0	35855	0
27002	CEBPA	19	33792368	c.593_595dup	p.(Leu198_Thr199insMet)	2198	45.1	0	24902	0
27002	CEBPA	19	33793199	c.214_226del	p.(Ala72Serfs*119)	1498	42.8	0	11832	0
27002	CSF3R	1	36932128	c.2339_2340del	p.(Leu780Hisfs*8)	1302	2.6	0	25613	0
27002	CSF3R	1	36933434	c.1853C>T	p.(Thr618Ile)	1422	1.8	3	24192	0.012
27002	GATA2	3	128202809	c.911C>A	p.(Pro304His)	1712	29	24	18844	0.127
27002	JAK3	19	17945969	c.1970G>A	p.(Arg657Gln)	1438	2.3	6	24112	0.024
27002	NRAS	1	115258744	c.38G>A	p.(Gly13Asp)	2104	2	0	25398	0
27002	NRAS	1	115258747	c.35G>C	p.(Gly12Ala)	2129	2.3	0	25257	0
27002	NRAS	1	115258747	c.35G>A	p.(Gly12Asp)	2129	13.5	2	25398	0.007
27002	WT1	11	32413568	c.1309_1331delinsT	p.(Lys437Serfs*6)	411	12	0	23300	0
27006	DNMT3A	2	25457243	c.2188C>T	p.(Arg730Cys)	1617	44.1	3347	31232	10.716
27006	FLT3	13	28608262	c.1764_1793dup	p.(Phe590_Tyr599dup)	1856	42.1	1	27295	0.003
28002	IDH2	2	209113112	c.419G>A	p.Arg140Glu		6	6	37907	0.015
28002	NPM1	5	170837546	c.863_864insCCTG	p.(Trp288Cysfs*?)		3	2	22273	0.008
28005	FLT3	13	28602329	c.2039C>T	p.(Ala680Val)	1582	27.9	25	30979	0.08
28005	MYC	8	128750680	c.217A>G	P.Thr73Ala	2763	38.2	2	35689	0.005
28005	MYC	8	128750684	c.221C>T	p.Pro74Leu	2728	9	3	36283	0.008
28005	NPM1	5	170837545	c.863_864insCATG	p.(Trp288Cysfs*?)	1665	28.2	1	22692	0.004
28005	PTPN11	12	112926249	c.1394C>G	p.(Ala465Gly)	1674	7.4	1	32666	0.003
28007	CBL	11	119149251	c.1259G>A	p.(Arg420Gln)	1108	54.6	7	24554	0.028
28007	NPM1	5	170837547	c.863_864insCTTG	p.(Trp288Cysfs*?)	1260	34.9	0	21288	0
28007	RAD21	8	117859785	c.1848del	p.(Tyr617Thrfs*?)	1493	40.5	2	23155	0.008

28007	SMC1A	X	53430550	c.2302C>T	p.(Arg768Trp)	916	39.8	6	28997	0.02
28008	NRAS	1	115258747	c.35G>A	p.(Gly12Asp)	680	22.7	30	45079	0.066
29003	ASXL1	20	31022441	c.1926_1927insG	p.Gly646TrpfsX12	2022	3	high noise	47919	<0.1
29003	DNMT3A	2	25467442	c.1178A>G	p.(Glu393Gly)	2397	46.6	14	50571	0.027
29003	FLT3	13	28592642	c.2503G>C	p.(Asp835His)	1732	47.1	0	38849	0
29009	FLT3	13	28592634	c.2508_2510del	p.(Ile836del)	1999	6.5	0	36200	0
29009	FLT3	13	28592640	c.2505T>A	p.(Asp835Glu)	1932	0.9	3	35736	0.008
29009	KIT	4	55599321	c.2447A>T	p.(Asp816Val)	1865	29.1	2	25447	0.007
29009	KRAS	12	25380275	c.183A>C	p.(Gln61His)	1233	1	0	31728	0
29009	MYC	8	128750687	c.224C>A	p.(Pro75His)	2358	41.5	3	36689	0.008
29009	NPM1	5	170837547	c.863_864insTATG	p.(Trp288Cysfs*?)	1400	36.8	0	27067	0
29009	NRAS	1	115258747	c.35G>A	p.(Gly12Asp)	1791	1	4	38806	0.01
29011	KRAS	12	25398285	c.34G>A	p.(Gly12Ser)	992	23.8	0	21883	0
29011	U2AF1	21	44524456	c.101C>A	p.(Ser34Tyr)	1938	5.6	0	24950	0
29012	TP53	17	7578518	c.412G>C	p.(Ala138Pro)	1996	57.8	22	38454	0.057
29013	EZH2	7	148504761	c.2218G>A	p.(Glu740Lys)	2104	2	21	25079	0.083
29013	KRAS	12	25398284	c.35G>A	p.Gly12Asp	1142	1	5	26384	0.018
29013	TET3	2	74328597	c.4682A>G	p.(Lys1561Arg)	2872	41.6	7233	31348	23.073
29013	WT1	11	32456559	c.332del	p.(Pro111Argfs*47)	1405	24	2	7648	0.026
30002	TP53	17	7577121	c.817C>T	p.(Arg273Cys)	1197	31.2	3305	25617	12.901
30002	TP53	17	7577520	c.761T>A	p.(Ile254Asn)	1257	9.1	4312	26674	16.165
30010	FLT3	13	28608262	c.1773_1793dup	p.(Tyr597_Glu598insAspValAspPheArgGluTyr)	2221	38.2	0	22752	0
30010	NPM1	5	170837543	c.860_863dup	p.(Trp288Cysfs*?)	1427	42.6	2	18086	0.011
30011	CEBPA	19	33793252	c.26dupC	p.(His10Alafs*84)	1479	54.8	4	7731	0.051
30011	DNMT3A	2	25470543	c.474del	p.(Ile158Metfs*6)	2092	50.1	6094	23235	26.227
30011	FLT3	13	28592628	c.2517T>A	p.(Asp839Glu)	1981	4.2	0	22841	0
30011	FLT3	13	28608341	c.1715A>C	p.(Tyr572Ser)	1985	2	0	14626	0
30011	NPM1	5	170837545	c.863_864insCATG	p.(Trp288Cysfs*?)	1990	44.2	0	17566	0
30014	ASXL1	20	31022697	c.2182G>T	p.(Glu728*)	1919	44.4	166	41222	0.402
30014	BCOR	X	39921391	c.4326+1G>A	p?	908	43.4	84	17597	0.477
30014	BCOR	X	39921430	c.4288G>T	p.(Glu1430*)	1031	35.5	110	18332	0.6
30014	DNMT3A	2	25459806	c.2021A>G	p.(Lys674Arg)	2261	49.2	84	17579	0.477
30014	DNMT3A	2	25468135	c.1085G>A	p.(Cys362Tyr)	2156	42	658	41775	1.575
30014	FLT3	13	28602340	c.2028C>A	p.(Asn676Lys)	1588	6.8	7	32639	0.021
30014	IDH1	2	209113113	c.394C>T	p.(Arg132Cys)	1822	45.1	170	33915	0.501
30014	KRAS	12	25378562	c.436G>C	p.(Ala146Pro)	2301	2.1	1	28334	0.003
30014	KRAS	12	25380279	c.179G>A	p.(Gly60Asp)	1451	3.6	1	31562	0.003
30014	TET2	4	106156072	c.973C>T	p.(Gln325*)	2471	1.1	2	40219	0.004
30015	FLT3	13	28608267	c.1759_1788dup	p.(Asn587_Glu596dup)	2947	46.5	0	22544	0
30015	NPM1	5	170837544	c.863_864insCCTG	p.(Trp288Cysfs*?)	2238	39.2	1	22557	0.004
30015	PHF6	X	133547940	c.673C>T	p.(Arg225*)	831	69.1	3	9140	0.032
30015	TET2	4	106194069	c.4530dupG	p.(Leu1511Valfs*67)	2042	1	0	23953	0
30015	TET2	4	106196213	c.4546C>T	p.(Arg1516*)	1925	40.4	18	28254	0.063

30015	TET2	4	106196285	c.4618C>T	p.(Gln1540*)	2058	46.7	146	40937	0.356
30018	DNMT3A	2	25457243	c.2188C>T	p.(Arg730Cys)	2363	41.4	7687	31045	24.76
30018	FLT3	13	28592634	c.2508_2510del	p.(Ile836del)	2477	26.7	0	33842	0
30018	NPM1	5	170837543	c.860_863dup	p.(Trp288Cysfs*?)	2336	37.5	0	24891	0
30018	NRAS	1	115258744	c.38G>A	p.(Gly13Asp)	2260	1.7	0	35798	0
30018	PTPN11	12	112888165	c.181G>T	p.(Asp61Tyr)	2040	7.2	3	29990	0.01
30018	SMC1A	X	53436052	c.1420C>T	p.(Arg474Cys)	769	57	5	19363	0.025
30018	SMC3	10	112343257	c.920T>C	p.(Leu307Pro)	1641	10.8	0	28616	0
30020	FLT3	13	28608218	c.1793_1837dup	p?	1681	38.1	0	23767	0
30020	WT1	11	32413556	c.1343A>G	p.(His448Arg)	151	5.9	0	23932	0
30021	NF1	17	29528494	c.1252dupA	p.(Ile418Asnfs*11)	578	9.3	0	19359	0
30021	NF1	17	29562678	c.3759_3760insCC	p.(Tyr1254Profs*13)	863	1	0	26727	0
30021	NF1	17	29587444	c.4425_4426insGGGA	p.(Ser1476Glyfs*6)	1039	2.2	0	23711	0
30029	FLT3 (ITD)	ND	ND	ND	ND	ND	ND	ND	ND	ND
30029	NPM1	5	170837543	c.860_863dup	p.(Trp288Cysfs*?)	997	41.8	0	16582	0
30031	DNMT3A	2	25457242	c.2189G>A	p.(Arg730His)	855	40.3	640	30979	2.065
30031	FLT3	13	28592642	c.2503G>C	p.(Asp835His)	892	39.7	0	30192	0
30031	NPM1	5	170837544	c.863_864insCCTG	p.(Trp288Cysfs*?)	741	40.9	4	19881	0.02
30031	TET2	4	106163991	c.3501G>T	p.(Arg1167Ser)	480	42.1	5	29362	0.017
30031	TET2	4	106164022	c.3532del	p.(Glu1178Lysfs*48)	594	39.8	3	26099	0.011
31009	DNMT3A	2	25457243	c.2188C>T	p.(Arg730Cys)	1136	42.9	7947	28571	27.814
31009	KRAS	12	25398284	c.35G>C	p.(Gly12Ala)	1032	1.4	0	22001	0
31009	NPM1	5	170837543	c.860_863dup	p.(Trp288Cysfs*?)	2905	35.9	6	21048	0.028
31009	NRAS	1	115258747	c.35G>A	p.(Gly12Asp)	1665	29.7	10	30539	0.032
31009	SMC3	10	112362692	c.3407C>T	p.(Pro1136Leu)	1406	46.2	7313	25842	28.298
31009	TET2	4	106158476	c.3378del	p.(Gln1127Asnfs*10)	1200	44.6	9	20544	0.043
32001	DNMT3A	2	25457243	c.2188C>T	p.(Arg730Cys)	1867	39.2	4231	29085	14.547
32001	FLT3	13	28592641	c.2504A>T	p.(Asp835Val)	1936	1.5	0	28016	0
32001	FLT3	13	28608275	c.1754_1780dup	p.(Ser585_Asp593dup)	1890	3.4	0	20383	0
32001	IDH2	15	90631934	c.263G>A	p.(Arg88Gln)	1984	37.1	2	34434	0.005
32001	NPM1	5	170837543	c.860_863dup	p.(Trp288Cysfs*?)	1532	30.2	0	17189	0
32002	FLT3 (ITD)	ND	ND	ND	ND	ND	ND	ND	ND	ND
32002	GATA2	3	128202758	c.962T>A	p.(Leu321His)	2794	4.5	8	36038	0.022
32002	MYC	8	128750684	c.221C>T	p.(Pro74Leu)	3064	4.2	10	36079	0.027
32002	NRAS	1	115258744	c.38G>A	p.(Gly13Asp)	2443	32.9	3	35234	0.008
32002	WT1	11	32417910	c.1086_1090dup	p.(Ser364Tyrfs*70)	1680	3.2	1	32671	0.003
32002	WT1	11	32417911	c.1079_1089dup	p.(Ser364Leufs*72)	1683	7	0	32509	0
32002	WT1	11	32417922	c.1082_1091dup	p.(Ala365Cysfs*6)	1704	4.4	0	33287	0
32003	BCOR	X	39922984	c.3622C>T	p.(Gln1208*)	2313	31.8	81	25507	0.317
32003	BCORL1	X	129162683	c.4152_4153insGG	p.(Leu1385Glyfs*71)	1303	31.4	88	27582	0.319
32003	RUNX1	21	36259154	c.256C>T	p.(Pro86Ser)	2900	30.1	111	32341	0.343
32007	TP53	17	7577538	c.743G>A	p.(Arg248Gln)	1658	3.5	2130	39124	5.444
32007	TP53	17	7578508	c.422G>A	p.(Cys141Tyr)	2200	61.4	2506	40476	6.191

32011	DNMT3A	2	25457242	c.2189G>A	p.(Arg730His)	2239	7.1	35	27214	0.128
32011	MYC	8	128750690	c.227T>G	p.(Leu76Arg)	2194	5.4	high noise	high noise	<0.1
32011	WT1	11	32456357	c.534del		1463	6.8	0	26662	0
32017	DNMT3A	2	25457243	c.2188C>T	p.(Arg730Cys)	1813	39.8	1537	30044	5.115
32017	FLT3	13	28592622	c.2523C>A	p.(Asn841Lys)	1778	22.3	1	30086	0.003
32017	FLT3	13	28592640	c.2505T>G	p.(Asp835Glu)	1731	7.8	0	30830	0
32017	NPM1	5	170837543	c.860_863dup	p.(Trp288Cysfs*?)	1069	30.4	0	21129	0
32017	NRAS	1	115258747	c.35G>A	p.(Gly12Asp)	1451	2.2	0	33962	0
32017	PTPN11	12	112926851	c.1483C>T	p.(Pro495Ser)	1661	2.3	3	24000	0.012
33001	DNMT3A	2	25457243	c.2188C>T	p.(Arg730Cys)	1643	47.3	977	20733	4.712
33001	NPM1	5	170837543	c.860_863dup	p.(Trp288Cysfs*?)	1858	37.9	0	14078	0
33001	PTPN11	12	112888156	c.172A>T	p.(Asn58Tyr)	1895	41.9	3	16247	0.018
33002	FLT3	13	28592620	c.2525A>G	p.(Tyr842Cys)	1690	1.8	0	15353	0
33002	FLT3	13	28592622	c.2523C>A	p.(Asn841Lys)	1673	1.6	3	15446	0.019
33002	KRAS	12	25380285	c.173C>T	p.(Thr58Ile)	1189	30	0	12909	0
33002	STAG2	X	123185018	c.1065T>G	p.(Tyr355*)	186	78.1	0	5315	0
33003	BCOR	X	39933073	c.1526G>T	p.Trp509Leu	937	1,8	2	17834	0.011
33003	DNMT3A	2	25464565	c.1948C>G	p.Leu650Val	1578	40.7	6673	39115	17.059
33003	EZH2	7	148506428	c.2084C>T	p.Ser695Leu	1486	52	2807	20244	13.865
33003	RUNX1	21	36164888	c.906_907insC	p.F303LfsX270	1258	39	2	44114	0.004
33003	TET2	4	106157380	c.2281delC	p.Pro761Leufs*52	3070	45	5253	33817	15.533
33003	TET2	4	106164061	c.3573G>T	p.Gln1191His	2214	0.4516711833785	4646	26141	17.772
33003	TET2	4	106180891	c.3921G>T	p.Arg1307Ser	1657	41	4	32621	0.012
33011	SRSF2	17	74732959	c.284_307del	p.(Pro95_Arg102del)	2277	39	54	31420	0.171
34003	DNMT3A	2	25457243	c.2188C>T	p.(Arg730Cys)	2097	47	6574	28579	23.002
34003	FLT3	13	28592642	c.2503G>T	p.(Asp835Tyr)	2144	5.1	4	29314	0.013
34003	FLT3	13	28608225	c.1738_1830dup	p.(Gln580_Leu610dup)	2766	5	0	25255	0
34003	NPM1	5	170837543	c.860_863dup	p.(Trp288Cysfs*?)	2018	37.1	142	22400	0.633
34003	PTPN11	12	112888166	c.182A>T	p.(Asp61Val)	2127	21.5	6	31170	0.019
34003	PTPN11	12	112888198	c.214G>A	p.(Ala72Thr)	2224	1.2	2	25003	0.007
34008	ATM	11	108216519	c.8468T>C	p.(Val2823Ala)	2517	36.9	0	19541	0
34008	DNMT3A	2	25457242	c.2189G>A	p.(Arg730His)	3684	41.2	3254	26460	12.297
34008	FLT3	13	28592642	c.2503G>T	p.(Asp835Tyr)	3729	1.2	3	29403	0.01
34008	NPM1	5	170837543	c.860_863dup	p.(Trp288Cysfs*?)	4355	35.1	0	20641	0
34008	NRAS	1	115258748	c.34G>A	p.(Gly12Ser)	3390	34.7	0	28671	0
34008	SMC3	10	112356174	c.1982G>C	p.(Arg661Pro)	1869	37	0	23414	0
34009	FLT3	13	28608237	c.1792_1818dup	p.(Glu598_Pro606dup)	2637	33.4	1	28702	0.003
34009	RUNX1	21	36252865	c.416G>A	p.(Arg139Gln)	2599	35	22	26804	0.082
35002	BCORL1	X	129148639	c.1891C>T	p.(Gln631*)	782	17.9	4	14682	0.027
35002	RUNX1	21	36252878	c.403A>G	p.(Arg135Gly)	2291	19.3	3	22805	0.013
35007	FLT3	13	28592640	c.2505T>G	p.(Asp835Glu)	2118	1.9	0	33418	0
35007	NRAS	1	115258747	c.35G>A	p.(Gly12Asp)	2194	43.1	7	33086	0.021
35011	DNMT3A	2	25469136	c.865del	p.(Val289Trpfs*210)	2991	29.2	16	27686	0.057

**35011**	FLT3	13	28592642	c.2503G>T	p.(Asp835Tyr)	2381	24.5	1	28960	0.003
**35011**	PTPN11	12	112888211	c.227A>C	p.(Glu76Ala)	2029	5.1	0	29171	**0**
**35011**	TP53	17	7576574	c.1003dupG	p.(Asp335Glyfs*?)	1384	1.3	0	1378	**0**
35018	BCOR	X	39932167	c.2428_2432delCGAGA	p.Glu811Thrfs*4		1	0	1643	**0**
**35018**	NPM1	5	170837542	c.859_860insTCTG	p.(Trp288Cysfs*?)		21	14	14703	**0.095**
35029	BCOR	X	39932295	c.2303del	p.(Leu768Trpfs*18)	3003	36	453	22717	**1.994**
**35029**	NRAS	1	115258744	c.38G>A	p.(Gly13Asp)	3203	26.1	491	28851	**1.701**
**35029**	PHF6	X	133551245	c.885del	p.(Ala296Profs*55)	1748	34.7	1979	17124	11.556

ND: not detected in NGS; ID:identity; CHR: chromosome number; VAF: variant allele frequency; CR1 : complete remission or complete remission with incomplete recovery; FLT3 (ITD): tandem duplication in FLT3 that were only detected with standard PCR at diagnosis

Supplementary table 5: patients and disease characteristics

ID	gender	age	WBC ($x10^9/L$)	WT1 MRD	KARYOTYPE	Lindsley ontogeny
1006	M	46	4	NEG	failure	secondary
1009	M	56	3	NEG	47,XY,+4[3]/46,XY[9]	Pan-AML
1010	F	40	10.7	na	normal	pan-AML
1011	M	34	34.4	NEG	47,XY,+8[10]/46,XY[6]	pan-AML
1013	M	50	1.9	NEG	47,XY,t(6;11)(27;23),+der(6)t(6;11)(q27;q23)[10]	de novo
1014	F	60	26.8	NEG	normal	de novo
1024	M	48	25.8	NEG	normal	de novo
2003	M	50	43.5	NEG	51,XY,+6, +8, +14, +15, +21 [15]	pan-AML
2004	F	58	1.6	NEG	normal	pan-AML
2005	M	44	249.7	POS	normal	de novo
2006	M	52	7	POS	46, XY, der(6) t(6;?) (p11;?), t(2;15) (p25;q24) [14] 46, XY [1]	pan-AML
2010	F	54	2	NEG	46, XX, del(7) (q31) [1] 47, idem, +8 [cp14]	pan-AML
3001	M	38	57.9	na	47,XY,+mar[3]/46,XY[20]	pan-AML
3007	F	48	30.5	na	46,XX,ins(4;8)(q35;q13q22)[2]/46,SS[18]	de novo
3008	F	58	1.3	na	46,XX,-D,+?19[3]/46,XX[5]	secondary
4001	F	40	160.6	NEG	normal	de novo
4003	F	20	2.9	na	47,XX,+8,t(9;11)(p22;q23)[20]	de novo
4004	M	22	4.68	NEG	46,X?[23]	pan-AML
4005	M	42	11.02	na	normal	de novo
4007	F	40	6.92	NEG	normal	secondary
4010	M	46	6.41	NEG	normal	de novo
4014	F	24	4.548	NEG	47,XX,t(3;5)(q26;q35),+8[7]/46,XX[7]	pan-AML
4015	F	50	7.44	na	46,X,-X,-2,-3,-10,add(21q),+4mars[2]/46,XX[3]	secondary
4020	M	56	2.2	NEG	normal	secondary
4021	F	52	2.18	POS	failure	de novo
4022	M	60	11.1	NEG	normal	de novo
4023	M	36	7.37	NEG	normal	de novo
4027	M	58	45.98	na	47,XY,+mar[5]/46,XY[18].ish7(D7Z1*2),8(D8Z1*2)[50],11q23(MLL*3)[4/60] nuc isk (D7Z1,D8Z1)*2[250],(MLL*3)[24/200]	secondary
4029	M	24	13.76	NEG	47,XX,+8[15]	pan-AML
4034	M	32	6.77	NEG	46,XY,t(3;5)(q25;q34)[21]	pan-AML
4038	F	52	96.57	NEG	46,XX,t(6;11)(q27;q23)[19].ish t(6;11)(5'MLL+;3'MLL+)[10]	de novo
4039	M	56	.93	NEG	45,X,-Y[5]/46,XY[15]	secondary
5008	F	30	2	POS	normal	pan-AML
5014	F	56	19.89	NEG	normal	de novo
5015	M	44	23.71	NEG	normal	pan-AML
5017	M	36	15	na	normal	pan-AML
5018	M	56	11.24	NEG	46,XY,t(1;2)(p36;p21)[21]	secondary
5021	M	52	1.96	na	normal	secondary
5022	F	42	45.32	NEG	1q trisomy	secondary
5023	F	54	27.97	na	normal	de novo
6002	F	42	9.33	NEG	normal	pan-AML
7003	M	34	14.8	NEG	46,XY,add(9)(p11) ou del(9)(p13)[4]/46,XY,der(9)add(9)(p2?2)add(9)(q?31)[17]	pan-AML
7006	M	28	19.9	na	normal	secondary
7012	M	40	178.6	NEG	normal	de novo
7013	F	28	4.4	na	48,XX,+4,+19[12]/46,XX[14]	pan-AML
7014	F	24	126	na	normal	pan-AML
7015	F	20	4.9	NEG	46, XX, del (4) (q?21q?32) [3] / 46, XX [19]	pan-AML
7016	F	42	51.1	NEG	normal	de novo
8002	M	34	66.6	POS	normal	pan-AML
8003	F	38	22.7	POS	normal	pan-AML
8004	M	48	13.8	POS	46,XY,t(6;9)(p23;q34)[17]/46,XY[3]	pan-AML
8005	M	50	8.4	NEG	46-47,der(2)t(1;2)(p13;q37),add(5)(q13)ordel(5)(q13q33),add(12)(p13),-22,+mar.ishr(7)(wcp7+),mar(wcp12+)[12]/47,idem,+i(8)(q10)[1]/47,idem,del(3)(q13)[5]	TP53
8012	M	34	71.2	NEG	normal	de novo
8017	M	18	2.9	NEG	47,XY,+8[8]/46,XY[12]	pan-AML
8020	M	60	6.3	na	normal	secondary
8022	M	42	6.4	NEG	47,XY,+i(4)(p10)[1]/46,XY[25]	de novo
8036	M	44	1.7	na	normal	pan-AML
10003	F	48	.92	NEG	normal	secondary
10004	F	54	1.18	POS	normal	pan-AML
10005	M	22	23.7	POS	normal	pan-AML
11016	F	54	65.3	na	normal	secondary

11019	F	38	57.36	NEG	normal	de novo
11033	M	18	1.9	na	45,XY, der(1)t(1;4)(p36;q22),-4,t(10;11)(p12;q21)[14]/46,XY[6]	de novo
12003	M	44	2.2	NEG	normal	secondary
12006	M	28	47.6	na	47,XY, +4[20]	secondary
12010	M	56	3.6	na	46,XY,add(6)(p?22), del (20)(q12)[8]/47,sl,+13[5]/46,XY[7]	pan-AML
12011	M	56	1.4	na	normal	pan-AML
12019	M	32	7.7	na	47,XY,+8,t(9;11)(p21;q23)[16]/46,XY[4]	de novo
12021	F	20	2.73	na	47,XX,+8[4]/46,XX[14]	de novo
12023	M	32	5.57	na	normal	pan-AML
12035	M	58	1.85	na	normal	secondary
13004	F	60	108.5	NEG	normal	de novo
13005	F	52	7	na	normal	de novo
13010	F	42	4	na	normal	pan-AML
13011	F	50	3	na	47,XX,-7[4]/90,idemx2[9]/46,XX[7]	pan-AML
13014	M	60	3.2	na	46,XY,t(1;3)(p36;q21)[19]/46,XY[2]	secondary
13021	M	42	15.8	NEG	46,XY,del(10)(q25)[4]/46,XY[16]	secondary
13054	M	28	5.65	na	normal	pan-AML
13064	M	40	11.31	na	normal	de novo
14002	M	28	5.75	na	normal	de novo
14005	F	28	2.25	NEG	47,XX,+8[12]/46,XX[3]	pan-AML
15002	M	36	33.9	NEG	normal	de novo
15005	F	42	7	NEG	normal	de novo
15012	M	42	8.9	na	normal	secondary
15016	F	48	2.9	na	normal	secondary
16007	M	48	20.6	POS	normal	de novo
16013	M	26	121	POS	47,XY,+8[6]/48,sl,+13[2]/46,XY[16]	secondary
16016	M	22	1.1	na	46,XY,inv(2)(q1?q2?3),der(?;10)t(10;q?11),-11,+?ace[14]/45,idem,add(16)(p1?2)[4]/46,XY[2]	pan-AML
16017	M	48	2.2	POS	47,XXY[7]	pan-AML
16019	M	44	4.7	NEG	44,XY,del(1)(q?21),-2,-3,add(4)(q3?1),del(5)(q13q33),del(7)(?p1?4),del(12)(p1?3),del(15)(q12),del(17)(q1?2),+mar[cp14]	TP53
16021	F	60	.9	NEG	normal	pan-AML
16023	M	28	56.47	na	47,XY,+8[17]	de novo
16029	F	46	147.2	na	normal	de novo
16037	M	46	2.2	na	normal	pan-AML
16038	M	20	2.11	NEG	46,XY,del(9)(q12q32)[24]/47,idem,+?8[2]/46,XY[5]	pan-AML
16040	F	60	8.71	na	normal	pan-AML
16041	M	56	5.58	NEG	normal	secondary
16043	F	24	11.01	NEG	normal	pan-AML
16044	F	22	57.33	na	47,XX,+8,t(9;11)(p22;q23)[18]/46,XX[2]	de novo
16046	M	50	120.3	NEG	normal	de novo
16050	F	52	1.93	NEG	normal	de novo
16052	F	42	14.13	na	46,XX,t(11;17)(q23;q?12),del(12)(p13)[20]	de novo
17005	M	58	1	NEG	43,XY,?dup(1)(p?p?),del(5)(q14q31),add(6)(p21),-14,add(14)(p11),-17,-18,add(19)(q13),-21,+22[8]/43,idem-9,+mar[4]/46,XX[9]	TP53
17012	F	46	62.4	NEG	normal	de novo
17014	F	48	2.2	na	56,XX,+X,del(5)(q14q34),+8,+9,+10,+11,+11,der(12)t(12;17)(p13;q25),+13,+14,add(17)(p13),18,+22,+2mars[20]	TP53
18006	M	36	30.38	NEG	normal	pan-AML
20002	M	34	2.9	na	normal	secondary
20010	F	50	9.1	NEG	normal	de novo
22003	F	56	1.54	NEG	normal	secondary
22004	F	44	8.99	POS	normal	pan-AML
22007	M	54	59.85	NEG	normal	secondary
22008	F	48	3.36	NEG	normal	pan-AML
22009	M	38	50	NEG	normal	pan-AML
23001	F	46	39.5	NEG	45,XX,inv(3)(q21;q26),7[13]	de novo
23004	M	48	7.5	na	45,XY,add(12)(p11),t(13;14)(q11;q31),-18,-20,+mar[4]/46,sl,+9[2]/46,sdl1,i(21)(q10)[2]/47,sdl1,+21[3]	pan-AML
23008	M	50	2.3	na	46,XY,add(7)(q11)[9]/46,XY[11]	pan-AML
23012	F	52	1.4	na	46, XX, -6, -10, +mar1, +mar2[4]/47, sl, +mar3[4]/46, XX[3]	pan-AML
23013	F	54	256	na	normal	de novo
23015	M	58	9.1	na	normal	de novo
23016	F	56	1.6	na	44,XX,-5,-16,add(17)(p13),add(22)(p13)[12]/44,sl,add(1)(q11),add(12)(q1?3),add(12)(p11),-17,+19[1]/46,XX[2]	TP53
23023	M	56	3.2	NEG	normal	secondary

23026	F	56	3.6	na	normal	pan-AML
23027	F	46	41.3	NEG	normal	de novo
23029	F	30	2.66	na	normal	secondary
24015	M	48	7.8	na	47,XY,+mar[2] / 45,XY, +3, der(8) t(8;13) (q23;?q31), -9,del(9) (p13-21),-13, t (15;17) (q14;p13)[13] / 46,XY[7]	TP53
24021	F	38	119	na	normal	pan-AML
24025	F	54	2	na	normal	de novo
24027	M	40	1.5	na	46,XY,ins(3;5)(q?21q?13q?23)[12]	pan-AML
24029	M	56	75.3	na	46,XY,?add(11)(q23)[20].isht(9;11)(p22;q23)	de novo
24032	M	48	15	na	46,XY,add(6)(p23)[17].ish t(6;9)(p23;q34)	pan-AML
24033	M	46	5.5	na	normal	secondary
24036	M	58	3	na	normal	secondary
24037	F	40	4.2	na	normal	pan-AML
24038	F	56	1.7	na	48,XX,+11,+ mar[2]	pan-AML
24040	F	60	2.2	na	normal	secondary
24042	M	52	49.6	na	normal	de novo
24043	F	50	109.9	na	46,X,add(X)(q27) or dic(X?),add(11)(q23),add(17)(q21)[10]	secondary
24044	M	60	96.6	na	normal	de novo
25002	M	20	9	NEG	46,XY,t(6;9)(p3;q34)[14]/46,XY[6]	pan-AML
25007	F	46	3.4	NEG	normal	secondary
25025	F	38	7.3	NEG	normal	pan-AML
25027	M	56	5	NEG	43-44,X,-Y,add(1)(p36),-2,der(4)t(4;10)(q21;q11),del(5)(q13q35),dup(7)(p13p15),dup(8)(q12q43),add(12)(p13),-16,-17,add(17)(p13),+m ,+m [cp14] / 46,XY [2]	pan-AML
26005	M	22	76.7	NEG	46, XY, inv(8) (p?q?), add (18) (q2?1), del (21) (q21q22) [6] / 46, XY [6] . ish inv(8) (p11q22) (ETO+, AML1-), add(18), del (21) (AML1+, ETO-) [3]. nuc (ETO, AML1x2) [100]	pan-AML
26007	F	44	10.9	NEG	normal	de novo
26009	M	48	2.88	na	normal	secondary
26010	F	36	52.72	NEG	normal	pan-AML
27002	F	40	11.2	NEG	47,XX,+21/46,XX	pan-AML
27006	M	32	53.7	na	normal	pan-AML
28002	F	52	1.9	NEG	normal	de novo
28005	F	20	17.2	NEG	normal	de novo
28007	F	26	2.5	NEG	normal	de novo
28008	M	42	7.2	NEG	normal	pan-AML
29003	F	54	99.14	na	48,XX,+8,t(9;11)(p22;q23)+der(9)t(9;11)[8]/49,sl,+12[9]/49,sl,+r[9]	secondary
29009	F	42	51.11	na	normal	de novo
29011	F	32	2.61	na	normal	secondary
29012	M	48	3.73	na	42,XY,der(2)del(2)(q22q32)?inv(2)(p24q32),-4,der(6)t(1;6)(q24;p24),-7,der(?)t(9;?)(p12;?),-10,+11,inv(11)(p1?5q?21)x2,-16,add(17)(p1?2),-18,-20,+mar[10]/43,sl,-add(17),?dic(17;20)(p11;q11),+mar[17]	TP53
29013	F	52	15.84	na	45,X,-X,?inv(11)(p11p15)t(11;16)(q12;p11),del(7)(p11),+22,~70dm	pan-AML
30001	M	24	11.92	na	normal	pan-AML
30002	M	38	1.8	POS	45,XY,der(5;17)(p10;q10),-7,+11,del(12)(q13q21)[10]	TP53
30010	M	50	93.6	NEG	normal	de novo
30011	F	42	5.33	NEG	normal	de novo
30014	M	58	9.89	na	normal	secondary
30015	M	54	56.85	NEG	normal	de novo
30018	M	38	18.3	NEG	46,XY,t(1;17)(p11;p11)?c[25]	de novo
30020	M	22	29.38	POS	47,XY,+8[26]/46,XY[4]	pan-AML
30021	F	30	1.86	NEG	48,XX,+4,t(10;11)(p12;q14),+17[10]/49,idem,+mar[2]/46,XX[8]	de novo
30029	F	50	36.97	NEG	normal	de novo
30031	M	36	6.59	na	49,XY,+8,+8[2]/49,sl,add(12)(p13)[12]/49,sl,add(3)(p25)[3]/49,sl,add(14)(p12)[3]/46,XY[5]	de novo
31009	F	42	15.9	na	47,XX,+8[3]/46,XX[17]	de novo
32001	F	36	23.6	NEG	normal	de novo
32002	M	36	7.9	NEG	47,XY,+8[12]/46,XY[10]	pan-AML
32003	F	56	.6	POS	normal	secondary
32005	M	50	.8	POS	46,XY,t(1;11)(q21;q23)[20]	de novo
32007	F	60	1.8	NEG	45,XX,-1,del(5)(q13q35),-7,t(12;17)(p11;q11),add(15)(p11),-20,+2mars[19]/46,XX[4]	TP53
32011	F	34	3.3	na	normal	pan-AML
32017	F	54	17	NEG	normal	de novo
33001	M	58	14.42	NEG	normal	de novo
33002	M	56	1.43	NEG	normal	secondary
33003	M	58	165.1	na	46,XY,t(5;15)(q35;q11),del(7)(q31[2]/46,sl,add(2)(qter)[2]/46,XY[2]	secondary
33011	F	36	16.67	na	46,XX,t(10;11)(p12;q23),add(17)(p11)[10]/46,XX[10]	secondary
34003	F	54	10.84	na	46,XX,del(9)(q21q23)[24]/46,XX,t(3;11)(q27;p12)[6]	de novo
34008	F	50	50.75	na	46,XX,del(15)(q21q24)[15]/46,XX[11]	de novo

34009	M	40	.53	NEG	47,XYYc?[34]	pan-AML
35001	F	58	1.79	na	47,XX,+1,der(1;7)(q10;p10),+mar[2]/51,sl,+8,+8,+14,+22<13>/50, sdl1,-mar[5]	pan-AML
35002	M	54	2.4	na	normal	pan-AML
35007	M	26	117.7	na	46,XY,del(1)(p32p35),t(9;11)(p21;q23)<20>	de novo
35011	M	52	11.43	na	normal	TP53
35018	M	44	6.18	NEG	47,XY,del(20)(q12)[11]/46,sl,del(12)(p11p12)[2]	secondary
35029	F	60	1.83	na	normal	secondary

M: Male ; F: FEMALE ; na : not available ; POS : *WT1* over-expression in CR ; NEG : no *WT1* overexpression in CR

Supplementary table 6: ontogeny according to NGS status

		**Total** (n=181)	**NGS^{other}** (n=53)	**NGS^{DTA}** (n=37)	**NGS^{NEG}** (n=91)	**P val**
**Lindsley ontogeny: N(%)**	De novo	63 (34)	10 (19)	24 (65)	29 (32)	P<0.0001
	Secondary	43 (24)	17 (32)	6 (16)	20(22)	
	TP53	10 (6)	7 (13)	0 (0)	3 (3)	
	Pan-AML	65 (36)	19 (36)	7 (19)	39 (43)	

NGS^{NEG} : no mutation detected in CR1; NGD^{DTA}: only mutations in *DNMT3A*, *ASXL1* or *TET2* detected in CR1; NGS^{other} : detection in CR1 of at least one mutation in another gene than *DNMT3A*, *ASXL1*, or *TET2*

Supplementary table 7: univariate analyses for prognosis

variable	CIR			RFS			OS		
	HR	CI	p-value	HR	CI	p-value	HR	CI	p-value
ELN17<br>Fav.<br>Int.<br>Adv.	 1<br>1.83<br>2.09	 -<br>0.93-3.6<br>1.04-4.18	0.095	 1<br>1.66<br>2.07	 -<br>0.91-3<br>1.13-377	0.0552	 1<br>1.7<br>2.86	 -<br>0.81-3.57<br>1.39-5.89	0.00848
age	1.02	1-1.05	0.0298	1.02	1-1.04	0.046	1.03	1-1.05	0.0138
CLARA	2.06	1.26-3.36	0.003	1.88	1.22-2.89	0.00361	1.86	1.13-3.06	0.0133
ECOG	1.27	0.88-1.85	0.21	1.37	0..99-1.91	0.0603	1.64	1.13-2.38	0.0114
WBC	1	1-1.01	0.607	1	1-1.01	0.314	1	1-1.01	0.055
N mut diag	0.98	0.88-1.1	0.779	1.03	0.94-1.14	0.528	1.06	0.95-1.19	0.316
NGS<br>NEG<br>DTA<br>other	 1<br>1.89<br>3.08	 -<br>0.93-3.83<br>1.72-5.52	0.00043	 1<br>1.83<br>2.49	 -<br>1.01-3.33<br>1.5-4.16	0.00134	 1<br>2.16<br>2.73	 -<br>1.09-4.28<br>1.49-4.98	0.00268
N mut in CR1*<br>0<br>1<br>Over 1	 1<br>1.67<br>3.21	 -<br>0.92-3.03<br>1.78-5.79	0.0012	 1<br>1.66<br>3.01	 -<br>0.91-3.04<br>1.79-5.03	0.00007	 1<br>1.72<br>3.5	 -<br>0.84-3.51<br>1.93-6.34	0.0000637

*analysis performed only in the 166 patients with at least 2 events at diagnosis. HR: hazard ratio; CI: confidence interval, CIR: cumulative incidence of relapse; RFS: relase free survival; OS: overall survival; fav: favorable; int intermediate; adv: advese; mut: mutations; WBC: intial white blood cell count; ; DTA : detection in CR1 of only *DNMT3A*, *ASXL1* or *TET2* mutations. Other: detection of at least one mutation in another gene than *DNMT3A*, *ASXL1 or TET2*

Supplementary table 8: number of persisting mutations in CR1 according to NGS MRD status in patients with two or more mutations at diagnosis

**Number of mut in CR**	**DTA (n=37)**		**Other (n=47)**	
	N patients	%	N patients	%
**1**	28	76	20	43
**2**	4	11	17	36
**3**	2	5	7	15
**4**	0	0	2	4
**5**	2	5	0	0
**6**	0	0	1	2
**7**	1	3	0	0

Mut : mutations ; DTA : detection in CR1 of only *DNMT3A*, *ASXL1* or *TET2* mutations. Other : detection of at least one mutation in another gene than *DNMT3A*, *ASXL1 or TET2*.

Supplementary table 9: multivariable analysis including WT1 and NGS MRD

	RFS			OS		
	HR	CI	P-value	HR	CI	P-value
NGS MRD POS	1.62	0.85 – 3.09	0.14	1.28	0.59 – 2.74	0.53
WT1 over-expressed in CR	3.21	1.55 – 6.67	0.0017	2.71	1.13 – 6.49	0.025
ELN2017 Int	1.01	0.46 – 2.22	NS	0.81	0.31 – 2.13	NS
ELN2017 Unfav	1.25	0.55 – 2.85	NS	1.65	0.67 – 4.11	NS

NS : not significant HR : Hazard ratio CI : 95% confidence interval ; RFS: relapse free survival; OS: overall survival; MRD: measurable residual disease

Supplementary table 10: multivariable analyses including NPM1 and NGS MRD in 67 NPM1 mutated patients

	CIR			RFS			OS		
	HR	CI	P-value	HR	CI	P-value	HR	CI	P-value
**NGS MRD POS**	3.37	1.09-10.39	0.035	2.33	0.96-5.7	0.063	2.3	0.81-6.54	0.12
**NPM1 MRD pos**	4.16	1.51-11.47	0.0059	3.55	1.53-8.25	0.0032	2.97	1.15-7.67	0.025
**ELN2017 Int**	1.09	0.41-2.9	0.87	1.23	0.53-2.85	0.62	1.62	0.63-4.13	0.31
**ELN2017 Unfav**	0.54	0.07-4.49	0.57	1.02	0.22-4.8	0.98	1.78	0.36-8.84	0.48

HR : Hazard ratio CI : 95% confidence interval ; CIR: Cumulative incidence of relapse; RFS: relapse free survival; OS: overall survival; MRD: measurable residual disease

Supplementary table 11: Patients outcome after allo-SCT in CR1

	CIR			RFS			OS		
	HR	CI	P-value	HR	CI	P-value	HR	CI	P-value
Allo-SCT (time dependent)	0.29	0.16 – 0.53	0.00006	0.50	0.29 – 0.85	0.011	0.75	0.41 – 1.39	0.37
NGS^{POS}	2.30	1.25 – 4.20	0.0071	1.97	1.17 – 3.33	0.011	2.15	1.18 – 3.93	0.013

HR : Hazard ratio CI : 95% confidence interval ; alloSCT : allogeneic hematopoietic stem cell transplant. CIR: Cumulative incidence of relapse; RFS: relapse free survival; OS: overall survival; NGS^{POS}: detection of at least one mutation in any gene in CR1.

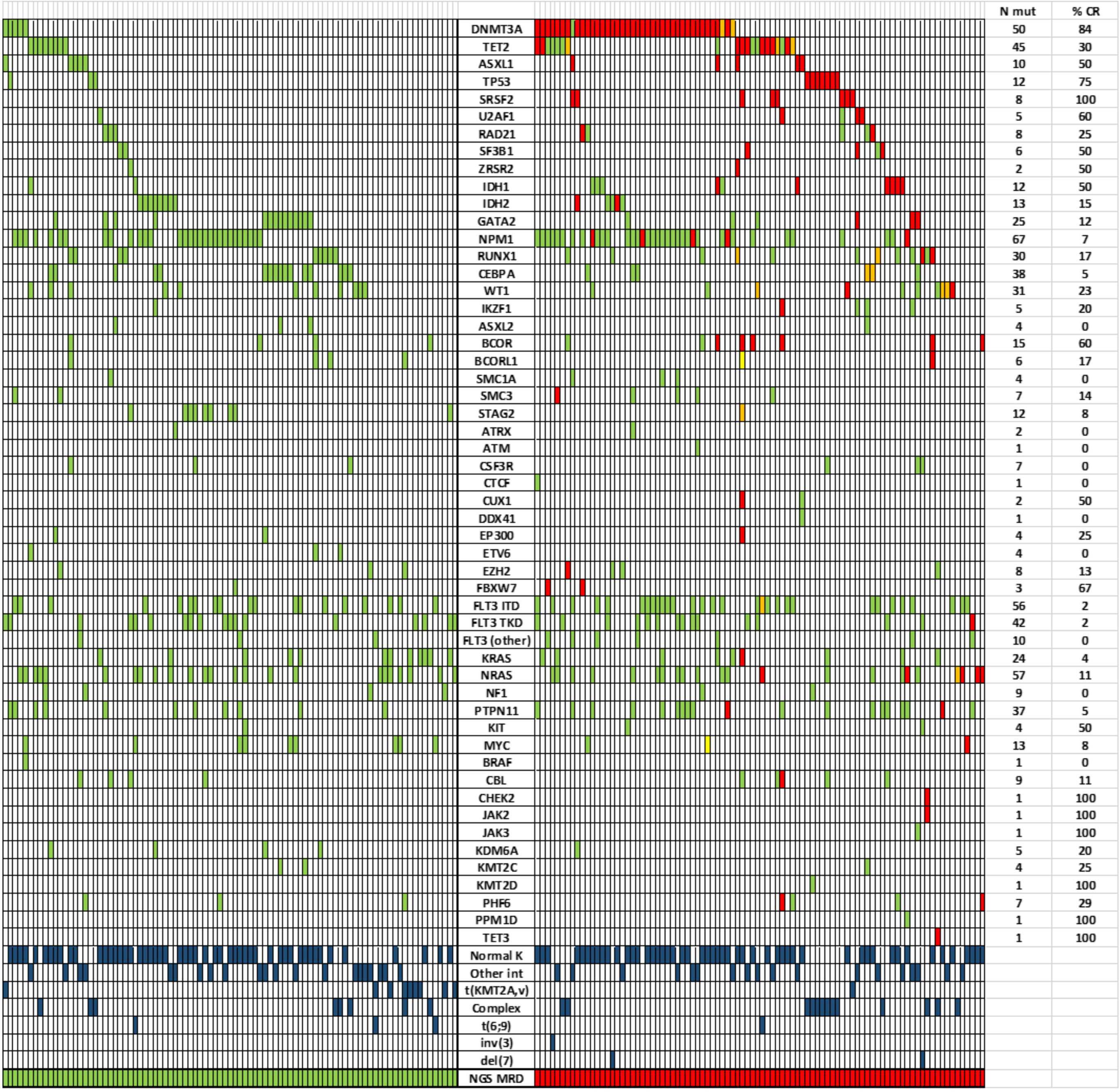

Supplementary figure 1: Comutation table at AML diagnosis and CR1.

Green boxes represent mutations detected at diagnosis and undetected in CR1. Red boxes represent mutation detected at both times. Orange boxes represent multiple mutations of the same gene detected at AML diagnosis with at least one mutation detected in CR1. Blue boxes represent chromosomal abnormalities at AML diagnosis.

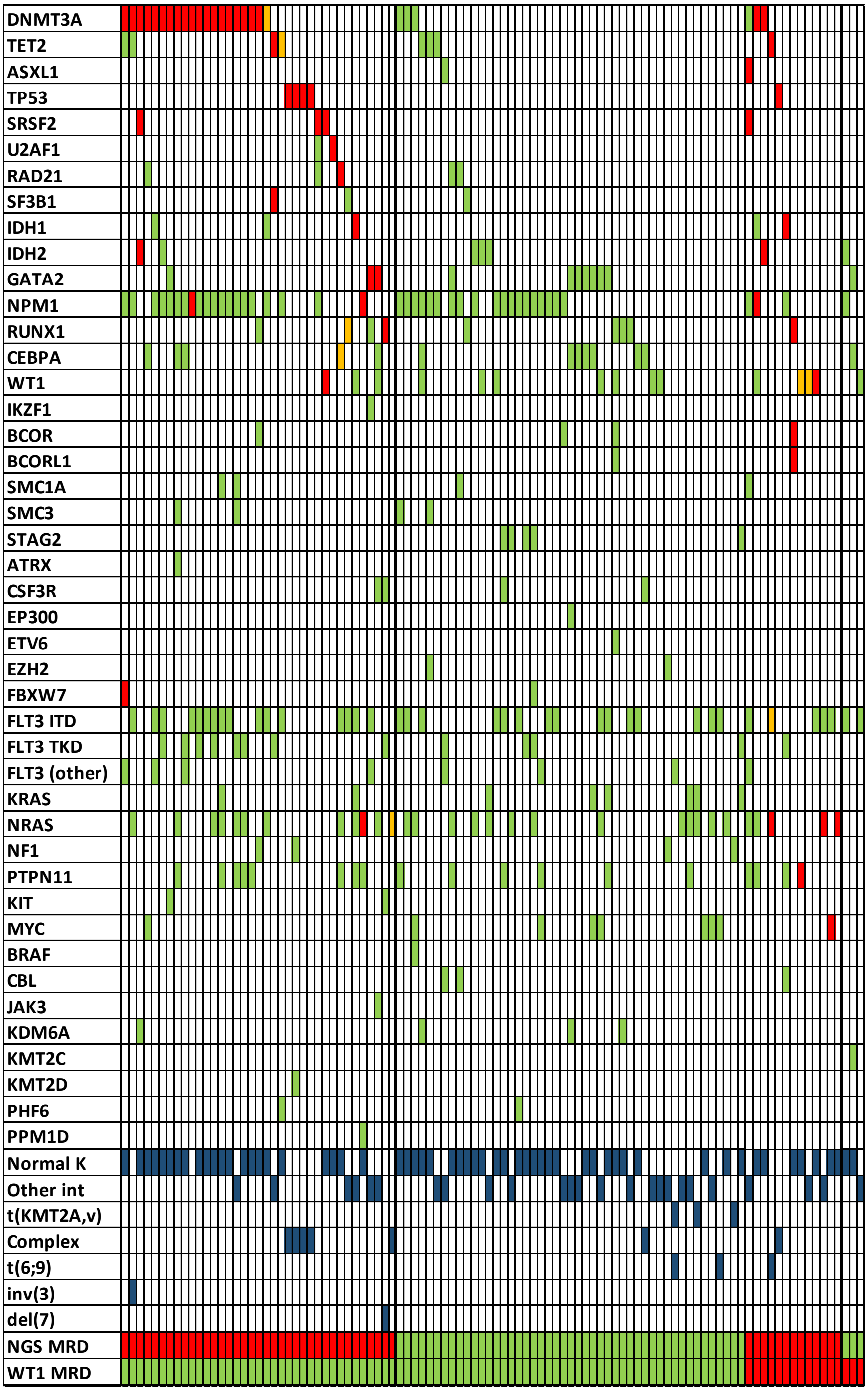

Supplementary figure 2: Comutation table in CR1 in the 100 patients with initial WT1 overexpression.

The color code is the same as supplemental figure 1. Patients with detectable NGS MRD are plotted in red in the NGS MRD line and patients without detectable mutations are plotted in green. Patients with persistent WT1 overexpression in CR1 are plotted in red in the WT1 MRD line, and patients without overexpression are plotted in light green.

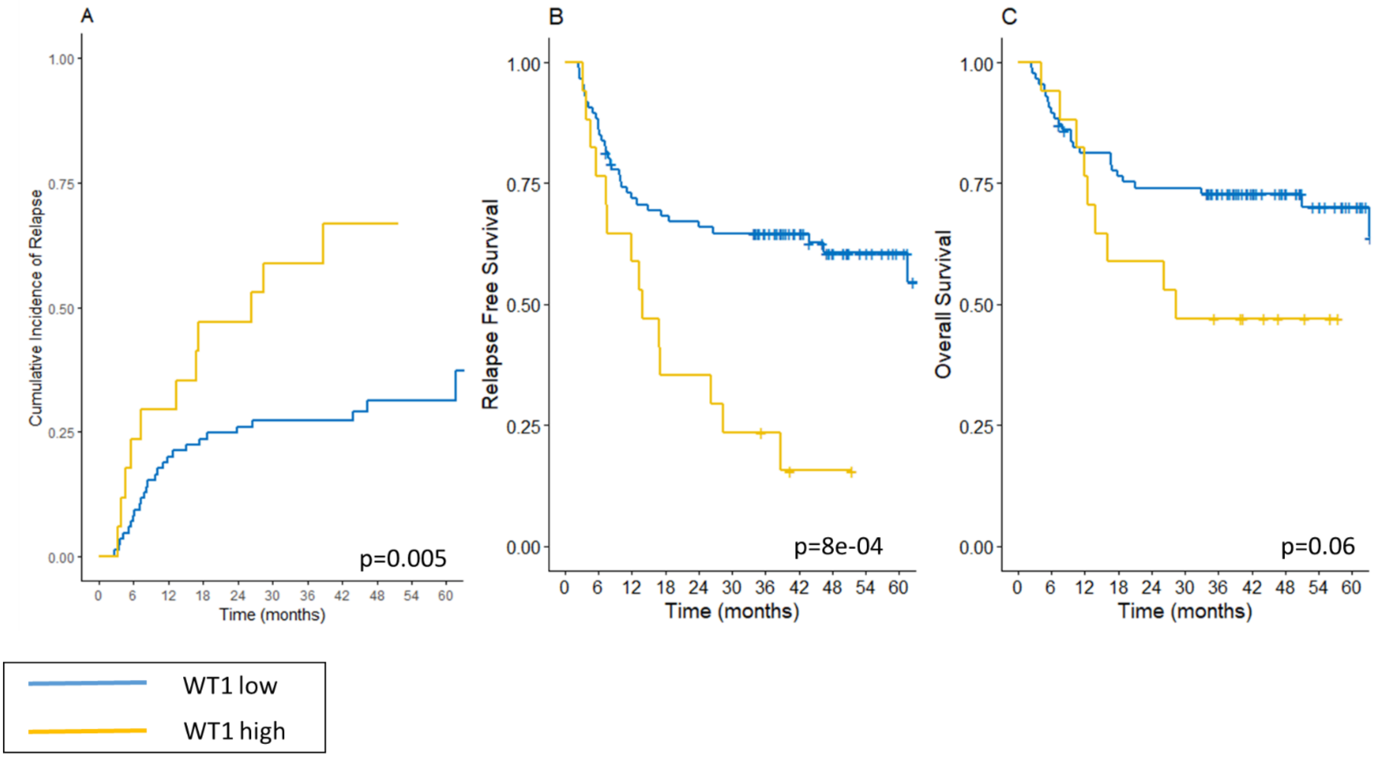

Supplementary figure 3: Prognosis according to *WT1* expression in CR1 in patients with initial *WT1* over-expression.

Patients with *WT1* over-expression in CR1 are represented in yellow and patients without *WT1* over-expression in CR1 are plotted in blue.

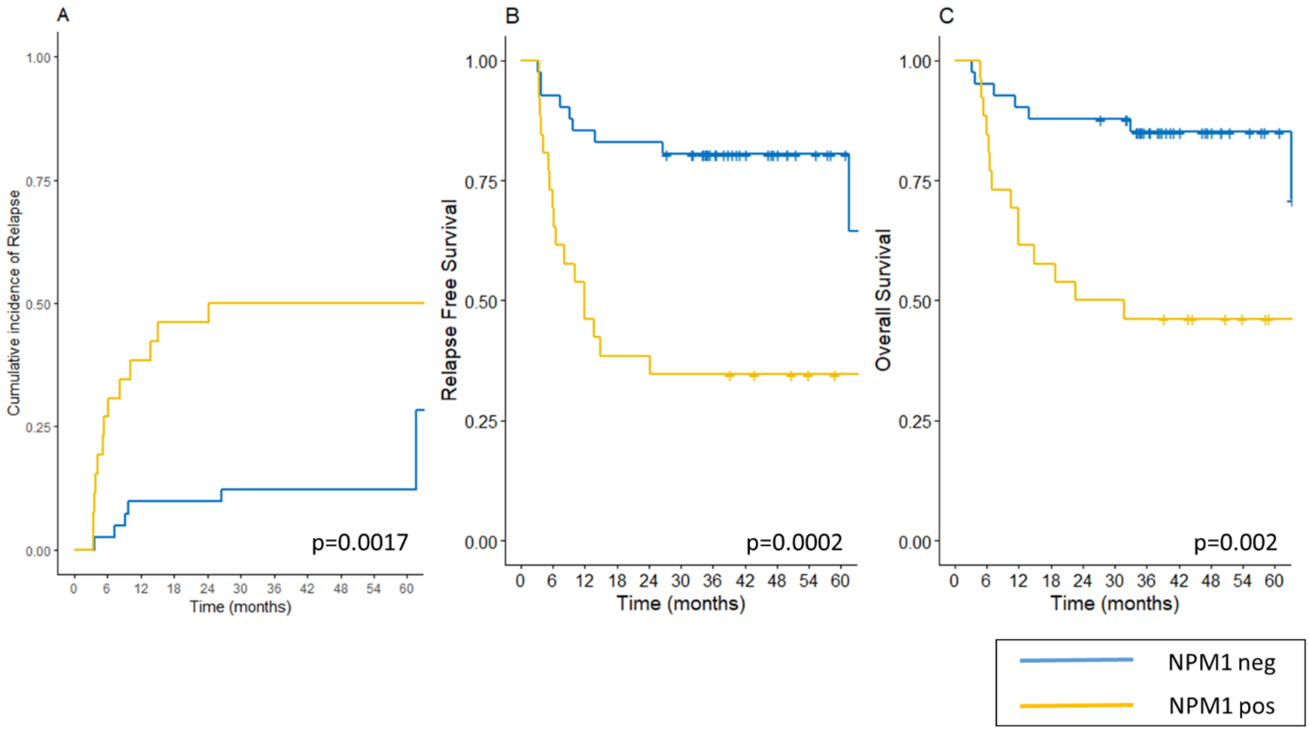

Supplementary figure 4: Prognosis according to *NPM1* MRD by error-corrected NGS.

The threshold for positivity is defined by detection of at last one consensus read.

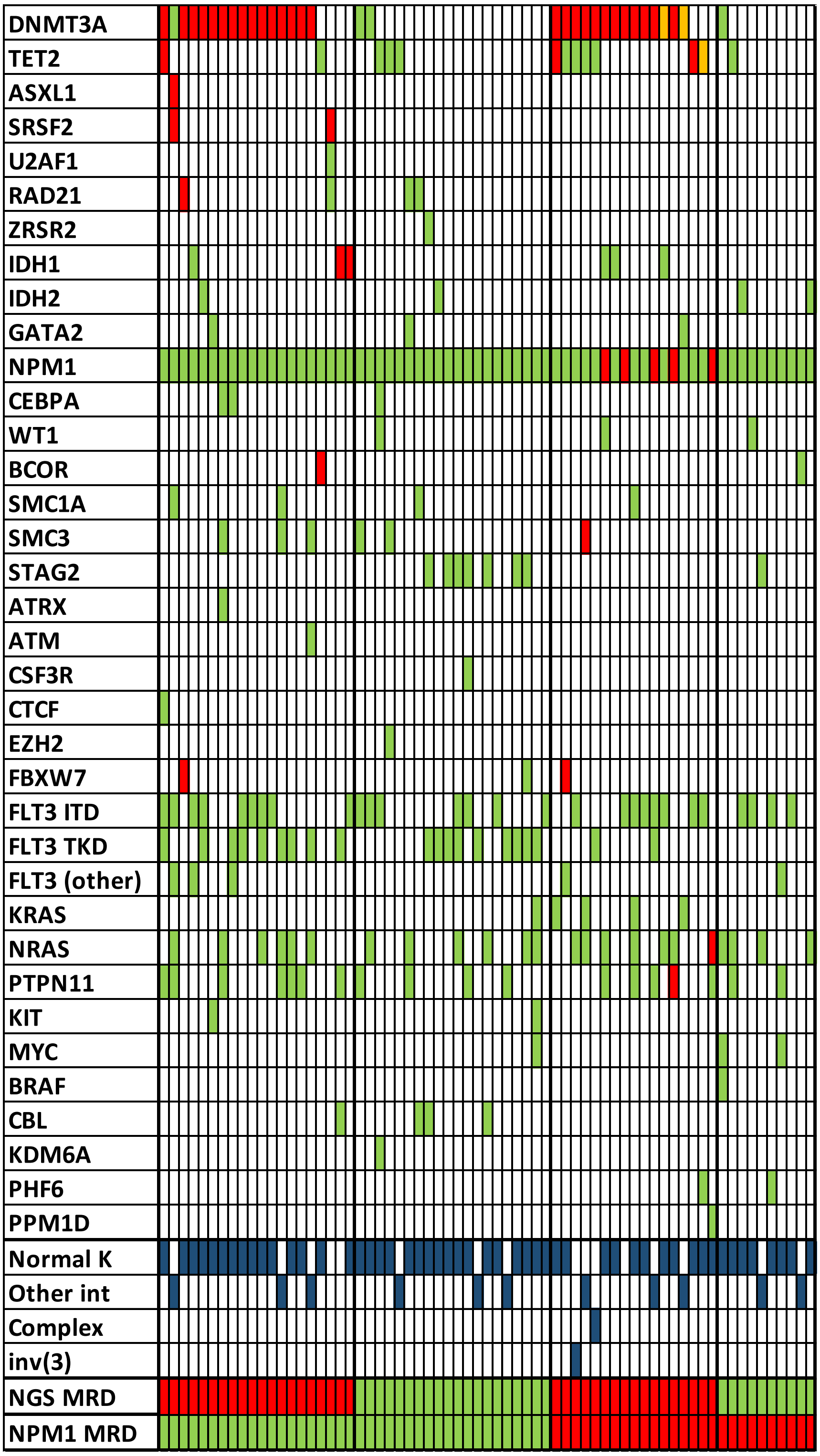

Supplementary figure 5: comutation table in CR1 in the 67 patients with initial *NPM1* mutation.

The color code is the same as sup figure 1. Patients with detectable NGS MRD are plotted in red in the NGS MRD line and patients without detectable mutations are plotted in green. Patients with persistent *NPM1* detection CR are plotted in red in the NPM1 MRD line, and patients without *NPM1* detection are plotted in green.

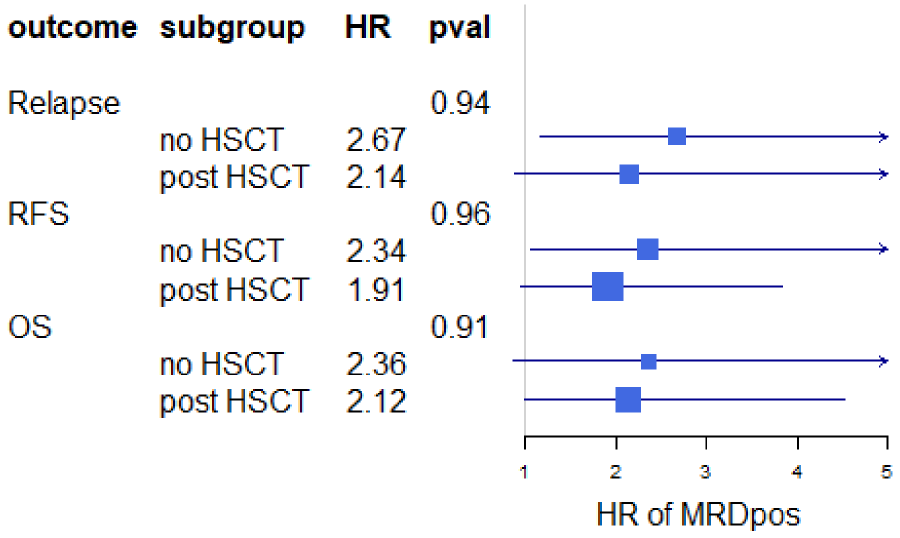

Supplementary figure 6: Interaction between allo-SCT and NGS-MRD for prognosis evaluation in the 127 intermediate or unfavorable ELN2017 patients.

RFS: Relapse free survival; OS: Overall survival. HSCT: allogeneic hematopoietic stem cell transplant.
